# Predictive Value of SpO2/RR Ratio for Short‐Term Intubation and Mortality Risks in Pre‐ED Patients With Acute Dyspnea Without Oxygen Therapy: A Retrospective Cohort Study

**DOI:** 10.1155/emmi/1372222

**Published:** 2026-06-30

**Authors:** Yuwei Tan, Sicheng Yuan, Panpan Wu, Xiaoqian Du, Hang Wang, Ge Guo, Lu Li, Li Dou, Tao Guo

**Affiliations:** ^1^ Emergency Department, (Jiangsu Hospital of Traditional Chinese Medicine), Affiliated Hospital of Nanjing University of Traditional Chinese Medicine, Nanjing, 210029, China

**Keywords:** acute dyspnea, emergency medicine, retrospective cohort study, SpO2/RR ratio (SR)

## Abstract

**Background:**

Dyspnea is a common and complex symptom in patients presenting to the emergency department, with multiple possible underlying diseases and a high risk of tracheal intubation or even death as the condition progresses. Therefore, an easily accessible, quick, and convenient index is needed to predict patient prognosis at emergency. A single measurement of peripheral capillary oxygen saturation (SpO_2_) or respiratory rate (RR) is not sufficiently accurate, while other related indices are relatively complex. This study investigated the predictive value of the SpO_2_/RR ratio (SR) in patients with acute dyspnea without oxygen therapy presenting to the emergency department.

**Methods:**

This single‐center retrospective analysis was conducted in the emergency department of Jiangsu Provincial Hospital of Traditional Chinese Medicine. Patients diagnosed with acute dyspnea without oxygen therapy who were presented to the emergency triage by the 120 ambulance were included. SR was calculated from the first vital signs obtained at the 120 ambulance real‐time transmission system for vehicles. Patients were divided into three groups based on terciles. Kaplan–Meier analysis, Cox regression models, and restricted cubic splines (RCS) were used to examine the associations between SR and clinical outcomes.

**Results:**

In total, 1339 patients were included. The rates of 7‐day intubation and 28‐day mortality differed significantly across SR groups (22.77%, 15.28%, 15.25%; *p* = 0.003) and (24.55%, 15.06%, 11.88%; *p* < 0.001), respectively. Cox regression and Kaplan–Meier analyses showed that lower SR was significantly associated with greater intubation and mortality risk (log‐rank *p* = 0.003, *p* < 0.001). Subgroup and sensitivity analyses supported these findings, with the effect more pronounced in patients with an RR of ≥ 30 breaths/min. RCS nonlinear analysis revealed L‐shaped associations between SR and 7‐day intubation and 28‐day mortality, with inflection points identified at 4.85 and 3.53.

**Conclusion:**

A lower SR was associated with increased 7‐day intubation and 28‐day mortality in emergency department patients with acute dyspnea without oxygen therapy. Inflection points of 4.85 and 3.53 suggest that SR may serve as a useful prognostic indicator for identifying high‐risk patients with acute dyspnea.

Yuwei Tan and Sicheng Yuan contributed equally to this work and both as co‐first authors.

## 1. Background

Acute dyspnea is the most common nontraumatic symptom encountered in emergency departments (EDs), functioning as the body’s alarm signal for hypoxia. It involves multisystem abnormalities related to respiration, circulation, neurology, and metabolism [1, 2]. Approximately 10% of prehospital emergency care patients and 4.0%–8.9% of ED patients present with dyspnea [3, 4]. This patient group often requires hospitalization and faces a poorer prognosis. In the 2012 CHARITEM study, patients whose primary symptom was acute dyspnea had significantly higher mortality rates than those presenting with chest pain or abdominal pain [5]. Research by Demoule et al. [6] also found that dyspnea was linked to an increased risk of intubation and elevated mortality.

Emergency medical services represent the first point of contact for patients receiving professional medical care and serve as a bridge to the ED. Identifying high‐risk patients with acute dyspnea after prehospital intervention remains a significant challenge for frontline emergency care. Because therapeutic measures are limited in the prehospital setting, a patient’s condition upon arrival at the ED—while ensuring life safety during transport—often provides a more accurate reflection of disease severity. Several scoring systems and biomarkers have been proposed for this patient group. Studies using the National Early Warning Score to predict outcomes in ED patients with acute dyspnea have shown that this score upon ED arrival strongly correlates with the need for intensive care unit admission [7]. Other research indicates that the perfusion index measured at ED admission can aid in triaging patients with acute dyspnea, with a cutoff value of 0.9 (sensitivity, 79.25%; specificity, 78.12%; positive predictive value, 66.7%; negative predictive value, 87.2%) [8]. However, these indicators are limited by the difficulty of obtaining them quickly.

Dyspnea refers to the subjective sensation of breathlessness, typically associated with tachypnea (respiratory rate [RR] > 20 breaths/min) and decreased peripheral capillary oxygen saturation (SpO_2_) [9]. While reduced SpO_2_ is a well‐recognized direct threat to life [10], tachypnea is often overlooked. Research by Koehler et al. [11] showed that in‐hospital mortality increases with rising RR. Accordingly, SpO_2_ (measured by pulse oximetry) and RR [11, 12]—as routine, noninvasive, continuous, and safe monitoring parameters—are integral to nearly all emergency early warning scoring systems [13–15]. This study was performed to examine the relationship between the SpO_2_/RR ratio (SR) and in‐hospital intubation rates as well as short‐term mortality in prehospital patients with acute dyspnea. It also assessed the potential value of the SR in supporting early intubation decisions and prognostic evaluation for these patients.

## 2. Methods and Materials

### 2.1. Data Source

This single‐center retrospective analysis was conducted in the ED of Jiangsu Provincial Hospital of Traditional Chinese Medicine, using data from the hospital’s electronic medical record system and 120 ambulance real‐time transmission system for vehicles. It included adult patients who were transported by the 120 ambulance to the ED for acute dyspnea from March 2023 to February 2025. The study was approved by the Institutional Review Board of Jiangsu Provincial Hospital of Traditional Chinese Medicine. The requirement for informed consent was waived because of the retrospective nature of the research.

### 2.2. Study Population and Definitions

Data were extracted from the hospital’s electronic medical record system and 120 ambulance real‐time transmission system for vehicles for patients who were transported by 120 to the ED and met the diagnostic criteria for acute dyspnea. The inclusion criteria were meeting the diagnostic criteria for acute dyspnea, age ≥ 18 years, no oxygen therapy or oxygen therapy time < 10 min, and relatively complete clinical data. The exclusion criteria were age < 18 years, oxygen therapy time ≥ 10 min, missing RR or SpO_2_ data, death on arrival at the ED, tracheal intubation on arrival at the ED, explicit refusal of intubation by the patient or family, and grossly incomplete study data.

### 2.3. Data Extraction

The extracted variables included demographic information, vital signs, final diagnoses, comorbidities, and basic laboratory parameters.

• Demographics: sex, age, race, and body mass index
• Vital signs: RR, SpO_2_, heart rate, temperature, systolic blood pressure (SBP), diastolic blood pressure (DBP), and mean arterial pressure. Data recorded upon the patient’s initial admission to the 120 ambulance. Specifically, after the 120 ambulance arrived at the patient’s location and the EMTs completed the initial assessment of the patient’s condition, they immediately connected the patient to a portable multiparameter monitor to collect real‐time vital signs. The first set of valid vital sign data was recorded once the monitor displayed stable readings (i.e., SpO_2_ and RR readings remained stable for ≥ 30 s without significant fluctuations), and this first set of data was immediately transmitted to the 120 ambulance real‐time transmission system for vehicles through a 4 G/5G network. We extracted the SpO_2_ and RR values from this first set of transmitted vital sign data to calculate the SR, ensuring that the data used reflected the patient’s baseline condition before any prolonged intervention during transport.
• Final diagnosis: pneumonia, asthma, pleural effusion, bronchitis, chronic obstructive pulmonary disease (COPD), pulmonary embolism, pulmonary fibrosis, lung cancer, cardiac insufficiency, myocardial infarction, aortic dissection, atrial fibrillation, hysteria, poisoning, trauma, and other diseases
• Comorbidities: hypertension, coronary heart disease, cardiac insufficiency, atrial fibrillation, cerebral infarction, Alzheimer’s disease, bronchitis, COPD, diabetes, renal insufficiency, cirrhosis, and malignant tumors
• Laboratory tests: The first laboratory values were obtained within 24 h of ED admission, including the white blood cell (WBC) count, hemoglobin (Hb), platelet count (PLT), high‐sensitivity C‐reactive protein (hs‐CRP), procalcitonin (PCT), blood urea nitrogen (BUN), creatinine, aspartate aminotransferase (AST), alanine aminotransferase (ALT), total bilirubin (Tbil), and albumin (ALB)
- Blood gas analysis: pH, lactate (Lac), PaO_2_, and PaCO_2_
- Coagulation: prothrombin time (PT), activated partial thromboplastin time (APTT), fibrinogen (FIB), and D‐dimer (DD)

### 2.4. Exposure

The SR was calculated as the ratio of SpO_2_ to RR. Patients were divided into three groups based on terciles.

### 2.5. Outcomes

7‐Day intubation: Whether the patient was intubated within 7 days (168 h) after arrival at the ED, either during the emergency stay or during hospitalization. The specific time of intubation was also recorded (measured from ED arrival).

Twenty‐eight‐day all‐cause mortality: survival status at 28 days (death or survival), measured from the time of ED arrival. The event of interest was defined as death occurring within 28 days.

### 2.6. Statistical Analysis

Variables with more than 30% missing data were excluded. Missing values for the remaining variables were imputed using multiple interpolations. Data imputation was performed for age, heart rate, SBP, DBP, temperature, pH, PaO_2_, PaCO_2_, Lac, hs‐CRP, WBC, PCT, Tbil, ALT, AST, Cr, ALB, BUN, Hb, PLT, PT, APTT, FIB, and DD. The imputed dataset was then divided into three groups based on terciles.

Continuous data were expressed as the mean ± standard deviation (SD) or median and quartile [M (Q1, Q3)] and were compared by the analysis of variance or the Kruskal–Wallis test as appropriate. Categorical data were reported as numbers and percentages [n (%)], and the comparisons were analyzed by the chi‐square test or Fisher’s exact test.

Kaplan–Meier survival analysis was conducted to estimate the incidence of 7‐day intubation and 28‐day mortality across SR groups, with the log‐rank test used to assess the statistical significance of differences between survival probabilities. Cox proportional hazards regression models were employed to estimate hazard ratios (HRs) and the corresponding 95% CIs for 7‐day intubation and 28‐day mortality. Potential confounders were adjusted in a stepwise manner. The low SR group was used as the reference for calculating HRs among the groups.

Restricted cubic splines (RCS) were used to test linearity and explore the shape of the dose–response association between SR and different endpoints of interest. The diagnostic/predictive value of the SR and related indices for outcome was analyzed using the receiver operating characteristic (ROC) curve. A *Z*‐test compared AUC values of the ROC curves for different indicators to determine whether differences in performances were statistically significant.

Subgroup analyses were conducted to evaluate potential effect modification according to sex (female or male), age (< 65 or ≥ 65 years), RR (< 30 or ≥ 30 breaths/min), SpO_2_ (> 93% or ≤ 93%), and the presence of medical conditions including diabetes, hypertension, cardiac insufficiency, bronchitis, COPD, and malignant tumors. For each factor, likelihood ratio tests were performed to assess interactions between SR and survival outcomes. Sensitivity analyses were conducted in the HRR group (≥ 30 breaths/min) and in individuals lacking RR or SpO_2_ data.

All statistical analyses were performed using R 4.2.3 software (Institute for Statistics and Mathematics, Vienna, Austria). A *p* < 0.05 (two‐sided) was considered statistically significant.

## 3. Results

### 3.1. Baseline Characteristics of Patients

**FIGURE 1:**
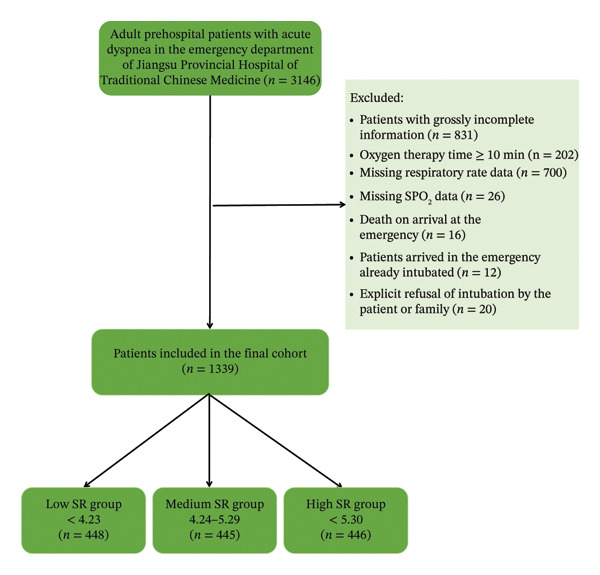
Flowchart of patient selection.

In total, 1339 patients with acute respiratory distress were enrolled in this study (Figure 1). The patients were divided into three groups based on terciles: 448 (33.46%) in Group 1, 445 (33.23%) in Group 2, and 446 (33.31%) in Group 3. The SR values of Groups 1 to 3 were 3.32 ± 0.67, 4.79 ± 0.29, and 6.09 ± 0.78, respectively. Comparison across the three groups showed that patients in Group 1 were more likely to experience 7‐day intubation and death within 28 days.

No significant differences in sex, SBP, DBP, pH, PaO_2_, PLT, diabetes, cerebral infarction, atrial fibrillation, cardiac insufficiency, Alzheimer’s disease, cirrhosis, renal insufficiency, or final diagnosis were observed across the SR groups. Patients in Group 1 presented with more severe respiratory distress upon admission, characterized by higher age, RR, heart rate, temperature, PaCO_2_, Lac, hs‐CRP, WBC, PCT, Tbil, ALT, AST, Cr, BUN, PT, APTT, FIB, and DD. Conversely, they had lower Hb and ALB. Group 1 also showed a higher prevalence of malignant tumors, hypertension, coronary heart disease, bronchitis, and COPD. Detailed baseline characteristics are presented in Table 1.

**TABLE 1:** Baseline characteristics of patients.

Variables	G1 (*n* = 448)	G2 (*n* = 445)	G3 (*n* = 446)	Statistic	*p*
SR	3.32 ± 0.67	4.79 ± 0.29	6.09 ± 0.78	F = 2250.21	**< 0.001**
Characteristic					
Age, years	72.68 ± 15.86	71.96 ± 16.96	67.77 ± 17.36	F = 11.19	**< 0.001**
BMI	22.59 ± 4.52	22.46 ± 4.80	22.53 ± 4.43	F = 0.10	0.907
Sex, *n (%)*				*χ* ^2^ = 0.73	0.694
Male	250 (55.80)	259 (58.20)	260 (58.30)		
Female	198 (44.20)	186 (41.80)	186 (41.70)		
Race, *n (%)*				—	0.710
Han ethnic group	398 (88.84)	384 (86.29)	395 (88.57)		
National minority	5 (1.12)	5 (1.12)	3 (0.67)		
Unknown	45 (10.04)	56 (12.58)	48 (10.76)		
Vital signs					
Temperature (°C)	36.74 ± 0.85	36.67 ± 0.83	36.56 ± 0.69	F = 5.40	**0.005**
Heart rate (times/min)	102.25 ± 49.53	92.06 ± 22.77	87.42 ± 21.95	F = 22.33	**< 0.001**
SBP (mmhg)	141.27 ± 35.23	138.23 ± 37.17	139.68 ± 35.00	F = 0.81	0.446
DBP (mmhg)	83.02 ± 22.86	79.52 ± 19.99	82.47 ± 38.14	F = 2.00	0.136
MBP (mmhg)	102.44 ± 25.24	99.09 ± 24.41	101.54 ± 31.49	F = 1.81	0.164
Laboratory values on admission					
PH	7.38 ± 0.12	7.39 ± 0.10	7.39 ± 0.10	F = 2.22	0.109
PaO_2_ (mmhg)	96.92 ± 42.36	98.75 ± 45.39	94.27 ± 41.59	F = 1.21	0.298
PaCO_2_ (mmhg)	39.83 ± 14.70	37.37 ± 12.25	37.68 ± 15.56	F = 3.95	**0.020**
Lac (mmol/L)	2.27 (1.50,4.10)	2.05 (1.30,3.30)	2.19 (1.44,3.20)	*χ* ^2^ = 9.07#	**0.011**
Hs‐CRP (mg/L)	21.53 (3.71,74.03)	7.32 (0.74,49.42)	3.28 (0.67,23.93)	*χ* ^2^ = 72.66#	**< 0.001**
WBC, × 10^9^	10.57 (7.57,13.85)	9.09 (6.88,12.35)	8.39 (6.17,11.54)	*χ* ^2^ = 36.35#	**< 0.001**
PCT (ng/mL)	0.21 (0.07,1.09)	0.15 (0.06,0.72)	0.11 (0.05,0.58)	*χ* ^2^ = 22.85#	**< 0.001**
Tbil (umol/L	17.00 (11.47,25.50)	16.87 (11.50,23.00)	17.91 (13.90,25.00)	*χ* ^2^ = 6.89#	**0.032**
ALT (U/L)	26.50 (20.00,44.00)	25.20 (18.00,39.00)	24.00 (18.00,36.00)	*χ* ^2^ = 10.39#	**0.006**
AST (U/L)	31.00 (23.00,52.00)	26.00 (21.00,38.00)	26.00 (20.00,39.00)	*χ* ^2^ = 29.34#	**< 0.001**
Creatinine (mg/dL)	87.65 (60.80,147.00)	82.90 (59.60,114.30)	79.55 (61.30,125.53)	*χ* ^2^ = 6.62#	**0.036**
ALB (g/L)	36.20 (31.08,40.62)	36.93 (31.80,41.50)	37.70 (33.15,42.10)	χ^2^ = 10.32#	**0.006**
BUN (mg/dL)	8.35 (5.97,14.22)	7.50 (5.60,11.70)	7.12 (5.10,10.49)	*χ* ^2^ = 24.29#	**< 0.001**
HB (g/L)	121.00 (100.00,137.00)	123.00 (102.00,138.00)	126.00 (107.00,143.00)	*χ* ^2^ = 8.13#	**0.017**
PLT, × 10^9^	193.50 (146.00,257.25)	199.00 (147.00,247.00)	196.00 (146.00,251.75)	*χ* ^2^ = 0.36#	0.833
PT (s)	14.70 (13.70,16.00)	14.30 (13.50,15.40)	14.10 (13.40,15.10)	*χ* ^2^ = 25.24#	**< 0.001**
APTT (s)	36.38 (33.10,40.02)	35.99 (32.10,40.50)	35.30 (31.72,38.50)	*χ* ^2^ = 15.00#	**< 0.001**
FIB (g/L)	3.99 (3.17,5.26)	3.67 (2.82,4.66)	3.24 (2.68,4.19)	*χ* ^2^ = 57.52#	**< 0.001**
DD (mg/L)	4.93 (1.47,85.66)	2.77 (0.86,64.27)	2.26 (0.52,47.95)	*χ* ^2^ = 29.44#	**< 0.001**
Final diagnosis, *n* (%)					
Pneumonia	82 (18.30)	84 (18.88)	98 (21.97)	*χ* ^2^ = 2.20	0.333
Asthma	38 (8.48)	43 (9.66)	40 (8.97)	*χ* ^2^ = 0.38	0.826
Pleural effusion	46 (10.27)	39 (8.76)	37 (8.30)	*χ* ^2^ = 1.15	0.564
Bronchitis	47 (10.49)	48 (10.79)	24 (5.38)	*χ* ^2^ = 10.18	**0.006**
COPD	49 (10.94)	28 (6.29)	25 (5.61)	*χ* ^2^ = 10.69	**0.005**
Pulmonary embolism	17 (3.79)	16 (3.60)	24 (5.38)	*χ* ^2^ = 2.10	0.351
Pulmonary fibrosis	14 (3.12)	16 (3.60)	26 (5.83)	*χ* ^2^ = 4.65	0.098
Lung cancer	18 (4.02)	17 (3.82)	16 (3.59)	*χ* ^2^ = 0.11	0.945
Cardiac insufficiency	27 (6.03)	30 (6.74)	30 (6.73)	*χ* ^2^ = 0.25	0.884
Myocardial infarction	16 (3.57)	15 (3.37)	7 (1.57)	*χ* ^2^ = 3.93	0.140
Aortic dissection	7 (1.56)	11 (2.47)	14 (3.14)	*χ* ^2^ = 2.40	0.301
Atrial fibrillation	24 (5.36)	15 (3.37)	19 (4.26)	*χ* ^2^ = 2.13	0.344
Hysteria	18 (4.02)	20 (4.49)	20 (4.48)	*χ* ^2^ = 0.16	0.923
Poisoning	20 (4.46)	21 (4.72)	15 (3.36)	*χ* ^2^ = 1.16	0.561
Trauma	4 (0.89)	9 (2.02)	13 (2.91)	*χ* ^2^ = 4.82	0.125
Other diseases	31 (6.92)	38 (8.54)	42 (9.42)	*χ* ^2^ = 1.89	0.389
Comorbidities, *n* (%)					
Hypertensive	257 (57.37)	237 (53.26)	214 (47.98)	*χ* ^2^ = 7.94	**0.019**
Diabetes	149 (33.26)	115 (25.84)	129 (28.92)	*χ* ^2^ = 5.98	0.050
Coronary heart disease	74 (16.52)	47 (10.56)	62 (13.90)	*χ* ^2^ = 6.74	**0.034**
Cerebral infarction	114 (25.45)	107 (24.04)	87 (19.51)	*χ* ^2^ = 4.86	0.088
Alzheimer’s disease	28 (6.25)	24 (5.39)	15 (3.36)	*χ* ^2^ = 4.13	0.127
Cirrhosis	17 (3.79)	12 (2.70)	13 (2.91)	*χ* ^2^ = 0.99	0.608
Renal insufficiency	27 (6.03)	23 (5.17)	19 (4.26)	*χ* ^2^ = 1.43	0.490
Malignant tumor	69 (15.40)	42 (9.44)	36 (8.07)	*χ* ^2^ = 13.90	**< 0.001**
Outcome					
7‐day intubation				*χ* ^2^ = 11.49	**0.003**
No	346 (77.23)	377 (84.72)	378 (84.75)		
Yes	102 (22.77)	68 (15.28)	68 (15.25)		
28‐day mortality				*χ* ^2^ = 27.33	**< 0.001**
No	338 (75.45)	378 (84.94)	393 (88.12)		
Yes	110 (24.55)	67 (15.06)	53 (11.88)		

*Note:* Bold values indicate the differences in indicators between different groups that were statistically significant.

### 3.2. Primary Endpoints

**FIGURE 2:**
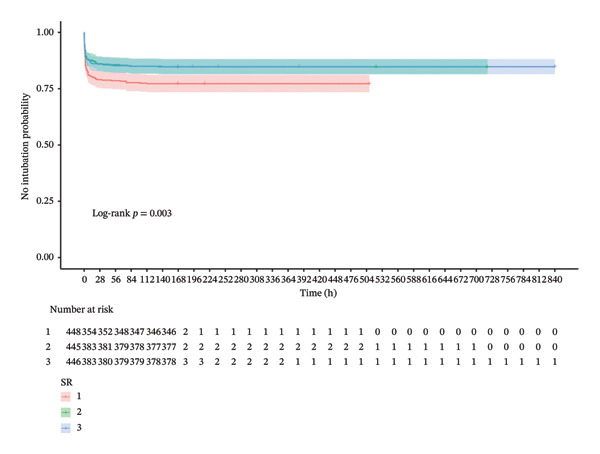
Kaplan–Meier survival curves showing intubation rates within 7 days across the three SR groups.

**FIGURE 3:**
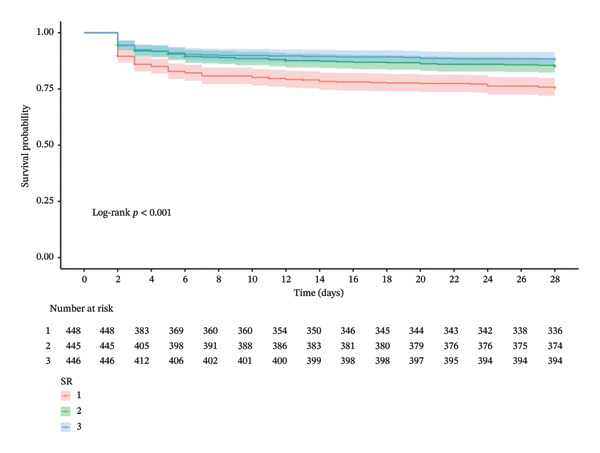
Kaplan–Meier survival curves showing 28‐day mortality across the three SR groups.

As shown in Figures 2 and 3, Kaplan–Meier survival curves were used to compare 7‐day intubation and 28‐day mortality rates across the three groups. The analysis revealed significant differences in both mortality and intubation rates among the groups. Specifically, Group 1 had substantially higher mortality and intubation rates than did the other groups (log‐rank *p* = 0.003, *p* < 0.001).

### 3.3. Cox Regression Models

We analyzed the correlation between SR and the primary outcomes using Cox regression models (Tables 2 and 3). When treated as a continuous variable, SR was independently associated with an increased risk of 7‐day intubation (HR, 0.88 [95% CI, 0.79–0.97]) and 28‐day mortality (HR, 0.81 [95% CI, 0.73–0.91]) in the fully adjusted model. Adjustment variables included all factors with *p* < 0.05 in the univariate Cox regression and LASSO for 7‐day intubation and 28‐day mortality, as detailed in Tables S1‐S2 and Figure S1‐S2. When considered as a categorical variable, in the fully adjusted model, Group 1 remained significantly associated with higher 28‐day mortality (Group 1 vs. Group 2: HR, 0.68 [95% CI, 0.49–0.93]; Group 1 vs. Group 3: HR, 0.62 [95% CI, 0.44–0.87]) and higher 7‐day intubation (Group 1 vs. Group 2: HR, 0.70 [95% CI, 0.51–0.96]; Group 1 vs. Group 3: HR, 0.71 [95% CI, 0.51–0.97]).

**TABLE 2:** Association between SR and 7‐day intubation.

Variables	Model 1		Model 2		Model 3	
	HR (95% CI)	*p*	HR (95% CI)	*p*	HR (95% CI)	*p*
SR	0.85 (0.76 ∼ 0.94)	**0.001**	0.83 (0.75 ∼ 0.92)	**< 0.001**	0.88 (0.79 ∼ 0.97)	**0.013**
SR group						
1	1.00 (Reference)		1.00 (Reference)		1.00 (Reference)	
2	0.64 (0.47 ∼ 0.88)	**0.005**	0.59 (0.43 ∼ 0.81)	**0.001**	0.70 (0.51 ∼ 0.96)	**0.025**
3	0.65 (0.48 ∼ 0.88)	**0.006**	0.63 (0.46 ∼ 0.86)	**0.004**	0.71 (0.51 ∼ 0.97)	**0.032**

*Note:* Model 1: crude. Model 2: adjusted for sex, race, age, and BMI. Model 3: adjusted for sex, race, diabetes, cerebral infarction, HR, Lac, hs‐CRP, and FIB. Bold values indicate the differences in indicators between different groups that were statistically significant.

Abbreviations: CI, confidence interval; HR, hazard ratio.

**TABLE 3:** Association between SR and 28‐day mortality.

Variables	Model 1		Model 2		Model 3	
	HR (95% CI)	*p*	HR (95% CI)	*p*	HR (95% CI)	*p*
SR	0.72 (0.65 ∼ 0.80)	**< 0.001**	0.73 (0.66 ∼ 0.82)	**< 0.001**	0.81 (0.73 ∼ 0.91)	**< 0.001**
SR group						
1	1.00 (Reference)		1.00 (Reference)		1.00 (Reference)	
2	0.59 (0.43 ∼ 0.79)	**< 0.001**	0.59 (0.43 ∼ 0.81)	**< 0.001**	0.68 (0.49 ∼ 0.93)	**0.017**
3	0.46 (0.33 ∼ 0.63)	**< 0.001**	0.49 (0.35 ∼ 0.68)	**< 0.001**	0.62 (0.44 ∼ 0.87)	**0.006**

*Note:* Model 1: crude. Model 2: adjusted for sex, race, age, and BMI. Model 3: adjusted for age, malignant tumor, SBP, Lac, hs‐CRP, WBC, ALB, BUN, and Hb. Bold values indicate the differences in indicators between different groups that were statistically significant.

Abbreviations: CI, confidence interval; HR, hazard ratio.

### 3.4. Detection of Nonlinear Relationship

**FIGURE 4:**
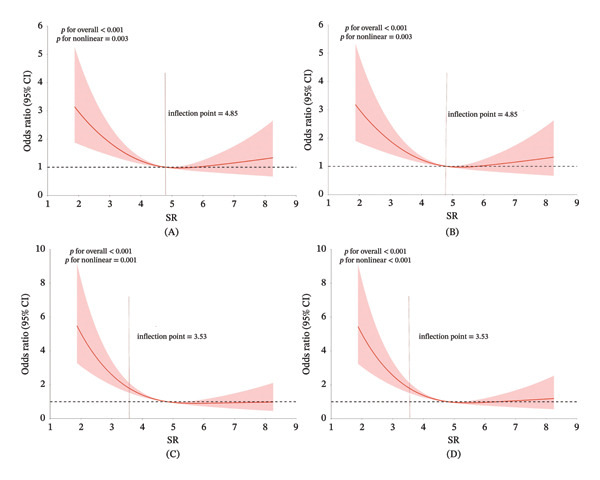
Dose–response association between SR and 7‐day intubation and 28‐day mortality based on restricted cubic spline analyses. Models (B/D) were adjusted for sex, age, race, and body mass index (kg/m^2^). Reference values were set at the median SR. Solid lines represent adjusted odds ratios and hazard ratios, shaded areas represent 95% confidence intervals, and vertical dashed lines indicate the inflection points.

RCS analysis revealed a nonlinear relationship between SR and 7‐day intubation and 28‐day mortality outcomes. As shown in Figure 4A–D, the 7‐day intubation rate and 28‐day mortality rate were all statistically significant (*p* for nonlinearity < 0.01). In the threshold effect analysis, SR inflection points were identified at 4.85 and 3.53 for 7‐day intubation and 28‐day mortality, respectively (see Tables 4 and 5). While the latter portions of the curves showed upward trends, the ORs and HRs for higher SR values were not significantly associated with intubation or mortality risk. The two‐piecewise linear regression models instead suggested more pronounced L‐shaped associations.

**TABLE 4:** Threshold effect analysis of 7‐day intubation (adjusted for sex, race, age, and BMI).

Outcome	Effect	*p*
Model 1 Fitting model by standard linear regression	0.83 (0.74–0.93)	**0.001**
Model 2 Fitting model by two‐piecewise linear regression		
Inflection point	4.85	
< 4.85	0.65 (0.52–0.81)	**<** **0.001**
≥ 4.85	1.13 (0.88–1.45)	0.337
*p* for likelihood test		**0.012**

*Note: p* values in bold indicate statistically significant differences.

**TABLE 5:** Threshold effect analysis of 28‐day mortality (adjusted for sex, race, age, and BMI).

Outcome	Effect	*p*
Model 1 Fitting model by standard linear regression	0.70 (0.62–0.78)	**<** **0.001**
Model 2 Fitting model by two‐piecewise linear regression		
Inflection point	3.53	
< 3.53	0.35 (0.20–0.61)	**<** **0.001**
≥ 3.53	0.87 (0.73–1.04)	0.132
*p* for likelihood test		**0.001**

*Note: p* values in bold indicate statistically significant differences.

### 3.5. Comparative Analysis of SR and Various Predictive Indicators Using ROC

To further evaluate the predictive performance of SR for outcomes, ROC analyses were conducted in comparison with SPO_2_, RR, age, inflammatory markers (PCT, WBC, hs‐CRP), and arterial blood gas analysis (PaO_2_, PaCO_2_, Lac, PH). It was ultimately found that, with regard to 7‐day intubation, the AUC for SR was 0.564 (0.516–0.607), which was lower than that for hs‐CRP but higher than that for the other indices, and the difference was statistically significant (*p* < 0.05). Regarding 28‐day mortality, the AUC for SR was 0.624 (0.586–0.661), which was lower than that for hs‐CRP but higher than that for the other indices, and the difference was statistically significant (*p* < 0.05). For details, see Figure S3‐S4 and Tables S3‐S4.

### 3.6. Subgroup Analysis

**FIGURE 5:**
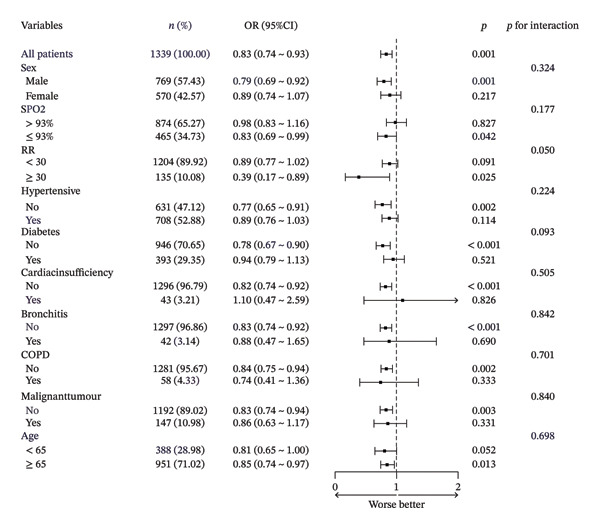
Forest plots of odds ratios for 7‐day intubation in different subgroups.

As shown in the subgroup analysis (Figure 5), a lower SR was significantly associated with higher risk in men; patients aged ≥ 65 years; those with an RR of ≥ 30 breaths/min; those with an SpO_2_ of ≤ 93%; and those without hypertension, cardiac insufficiency, bronchitis, COPD, diabetes, or malignant tumors.

**FIGURE 6:**
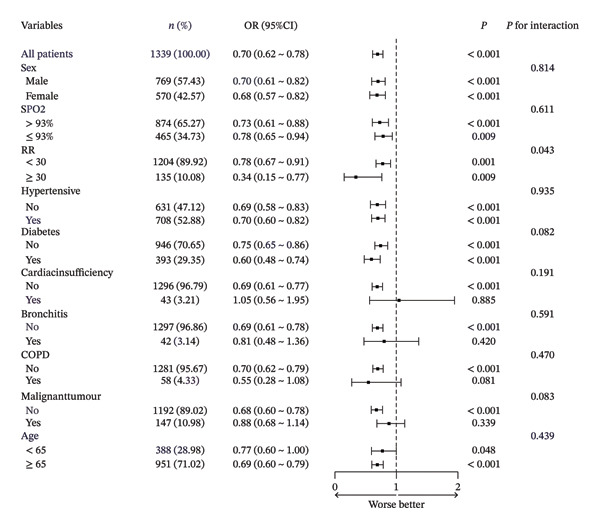
Forest plots of odds ratios for 28‐day mortality in different subgroups.

Similarly, in stratified analyses of 28‐day mortality (Figure 6), SR again showed stronger predictive value in the RR subgroups (RR < 30 breaths/min: OR, 0.78 [95% CI, 0.67–0.91]; RR ≥ 30 breaths/min: OR, 0.34 [95% CI, 0.15–0.77]; *p* for interaction = 0.043).

### 3.7. Sensitivity Analysis

Subgroup analyses identified an interaction effect of RR on the study endpoints. To further assess this effect, patients were divided into HRR and NRR groups, and the association between SR and endpoints was analyzed separately within these subgroups. In total, 135 patients were included in the HRR group. These patients were further divided into three groups based on terciles. Detailed baseline characteristics are presented in Table S5. As shown in Figure S5‐S6, Kaplan–Meier survival curves were used to compare 7‐day intubation and 28‐day mortality rates across the three groups. No substantial differences in mortality or intubation rates were observed. Specifically, the mortality and intubation rates in Group 1 were not significantly higher than those in the other groups (log‐rank *p* = 0.228, *p* = 0.051). We analyzed the correlation between SR and the primary outcomes using Cox regression models (Tables S6 and S8). When treated as a continuous variable, SR was independently associated with an increased risk of 7‐day intubation (HR, 0.49 [95% CI, 0.26–0.92]) and 28‐day mortality (HR, 0.42 [95% CI, 0.22–0.80]) in the fully adjusted model. Adjustment variables included all factors with *p* < 0.05 in the univariate Cox regression for 7‐day intubation and 28‐day mortality, as detailed in Tables S7 and S9. When considered as a categorical variable, however, SR was not independently associated with an increased risk of either 7‐day intubation or 28‐day mortality in the fully adjusted model.

RCS analysis further revealed a linear relationship between SR and 7‐day intubation and 28‐day mortality outcomes (*p* for nonlinearity > 0.05). As shown in Figure S7A–D, threshold analysis was not required.

In this study, owing to the significant number of patients lacking SpO_2_ or RR data, we compared the baseline characteristics of the 726 patients with missing SpO_2_ or RR values with those of the 1339 included patients to further validate the correlation between SR and outcomes. Statistical differences were observed between the two groups in race, pH, and AST (*p* < 0.05), whereas no significant differences were found for other characteristics (Table S10). Additionally, 726 patients were incorporated into the primary analysis, bringing the total number of patients included to 2065. These patients were further divided into three groups based on the tertiles. The detailed baseline characteristics are presented in Table S11. We analyzed the correlation between SR and primary outcomes using Cox regression models (Tables S12 and S14). SR was independently associated with an increased risk of 7‐day intubation and 28‐day mortality in the fully adjusted model. The adjustment variables included all factors with *p* < 0.05 in the univariate Cox regression and LASSO for 7‐day intubation and 28‐day mortality, as detailed in Tables S13‐S15 and Figure S8‐S9.

## 4. Discussion

To our knowledge, this is the first retrospective clinical study to use SR to identify high‐risk patients with acute dyspnea in the pre‐ED. We evaluated the predictive value of SR measured at triage for in‐hospital clinical deterioration, including the 7‐day intubation rate and 28‐day mortality. In this study, we calculated the 120 ambulance immediate SR of patients presenting to the ED with dyspnea and categorized them into three groups based on tertiles: high SR (> 5.30), intermediate SR (4.24–5.29), and low SR (< 4.23). The results showed significant differences in 7‐day intubation and 28‐day mortality across the three SR groups. A lower SR was significantly associated with a higher risk of both intubation and mortality.

Currently, many studies focus on the P/F ratio (PaO_2_/FiO_2_), S/F ratio (SpO_2_/FiO_2_) [16–22], ROX index (SpO_2_/FiO_2_/RR) [23–27], and VOX index (SpO_2_/FiO_2_/Vt) [28–31], most of which have been conducted in hospitalized patients in respiratory and intensive care units. These indices are primarily used to predict the need for intubation in patients with respiratory failure or to guide weaning and extubation. However, SR may be more suitable for use in the ED setting because most prehospital patients have not received oxygen therapy or have only received low‐flow oxygen for a short time, making FiO_2_ difficult to determine. By contrast, SR is easier to obtain. The SR integrates two core parameters—oxygenation efficiency and respiratory compensation—enabling early and rapid assessment of dyspnea.

The RCS curves demonstrated a clear L‐shaped nonlinear association of SR with 7‐day intubation and 28‐day mortality, with inflection points at 4.85 and 3.53, respectively. Below these thresholds, declining SR was accompanied by a marked elevation in short‐term intubation and mortality risk; beyond the inflection points, the risk curve plateaued, and higher SR no longer conferred additional prognostic information. These two cutoff values could serve as clinically actionable stratification boundaries to rapidly identify high‐risk acute dyspnea patients in the pre‐ED setting.

To further explore the intrinsic link between SR and the risk of intubation or mortality, we also observed that, compared with sex, age, SpO_2_, and comorbidities, SR showed greater predictive value for 7‐day intubation, in‐hospital intubation, and 28‐day mortality among patients with an RR of ≥ 30 breaths/min. This finding further supports the utility of SR in screening patients with acute dyspnea, as it may help exclude some patients with compensatory tachypnea while accurately identifying those at high risk. For individuals with missing data (particularly SpO_2_ and RR), a significant proportion of patients in the initial cohort were excluded. In the sensitivity analysis, we included this population in the primary analysis and found that the conclusions remained robust.

In terms of predictive efficacy, we found that the AUC of hs‐CRP was slightly higher than that of SR. However, hs‐CRP is a laboratory‐based biomarker that can only be tested upon arrival at the hospital, whereas SR is more readily available in prehospital settings, offering faster testing speed and convenience for early and rapid assessment of dyspnea. Future studies could consider combining both biomarkers for comprehensive evaluation.

This study has several limitations. First, it is a retrospective analysis and does not include detailed documentation of the reasons for intubation. Although most intubations occurred within 7 days, late intubation may have been influenced by confounding factors, such as fluid overload. Meanwhile, the decision to perform intubation is influenced by clinicians’ judgment, local diagnostic and treatment protocols, and resource availability. This endpoint is semisubjective rather than purely objective. Second, the study was conducted at a single center. Because of regional characteristics, referral patterns, hospital specialization, and admission criteria, the findings may lack generalizability. Larger multicenter studies are needed to confirm the predictive role of SR in patients with acute dyspnea. Third, during prehospital care, oxygen is most commonly delivered via masks or simple devices without flow measurement, making FiO_2_ unavailable. Consequently, SR values were not adjusted for inspired oxygen concentration, which may mask the true severity of illness in some patients receiving high‐concentration oxygen. These factors limit the generalizability of the research findings. Fourth, owing to missing data (particularly for SpO_2_ and RR), a significant proportion of patients in the initial cohort were excluded. In emergency and prehospital care settings, patients with more severe conditions (e.g., those requiring urgent intervention) or those with milder conditions (those receiving less monitoring) may have been disproportionately excluded. This raises concerns regarding selection bias, necessitating rigorous prospective studies for validation. Fifth, owing to the lack of records regarding consciousness levels and specific oxygen therapy protocols in this study, it is difficult to compare the results with traditional indicators (such as ROX, NEWS, and MEWS), which represents an area requiring further refinement in future research. Although we compared SR with SPO_2_, RR, age, inflammatory markers, and arterial blood gas analysis parameters, the AUC value of SR was higher than that of other indicators. However, the AUC values for all parameters were below 0.7, and no comparison was made with the composite severity score. These findings limit the clinical utility of SR, necessitating larger‐scale prospective studies to validate its efficacy. Finally, common causes of acute dyspnea, such as COPD and heart failure, affect the baseline RR and SpO_2_. These differences may influence the predictive value of the RR for clinical outcomes and the determination of cutoff values in specific subgroups. Future studies should further explore disease‐specific cutoff values for patients with particular comorbidities (e.g., COPD and heart failure).

## 5. Conclusion

The pre‐ED SR is associated with short‐term intubation and mortality risk. Its use could aid in the early recognition of high‐risk patients without oxygen therapy in the ED triage, but its discriminative performance is modest and not clearly superior to SpO_2_ alone, and prospective validation is required before clinical implementation.

**Nomenclature**
ED: Emergency department
RR: Respiratory rate
SpO_2_
: Peripheral capillary oxygen saturation
SR: SpO2/RR ratio
ICU: Intensive care unit
RCS: Restricted cubic splines
SBP: Systolic blood pressure
DBP: Diastolic blood pressure
MBP: Mean blood pressure
HR: hazard ratio
WBC: White blood cell
HB: Hemoglobin
PLT: Platelet count
Hs‐CRP: High‐sensitivity C‐reactive protein
PCT: Procalcitonin
COPD: Chronic obstructive pulmonary disease
BUN: Blood urea nitrogen
AST: Aspartate aminotransferase
ALT: Alanine aminotransferase
ALB: Albumin
Lac: Lactate
PT: Prothrombin time
APTT: Activated partial thromboplastin time
FIB: Fibrinogen
DD: D‐dimer
IQR: Interquartile range
CI: Confidence interval

## Author Contributions

Yuwei Tan: writing–review and editing, writing–original draft, methodology, investigation, formal analysis, data curation, and conceptualization. Sicheng Yuan: writing–review and editing, writing–original draft, methodology, investigation, formal analysis, and data curation. Panpan Wu, Xiaoqian Du, Hang Wang, Ge Guo, and Lu Li: methodology and data curation. Li Dou: writing–review and editing and methodology. Tao Guo: writing–review and editing, supervision, resources, project administration, methodology, and conceptualization.

## Funding

This research did not receive any specific grant from funding agencies in the public, commercial, or not‐for‐profit sectors.

## Ethics Statement

The study was approved by the Institutional Review Board of the Affiliated Hospital of Nanjing University of Traditional Chinese Medicine (Jiangsu Hospital of Traditional Chinese Medicine). This study was performed in line with the principles of the Declaration of Helsinki.

## Conflicts of Interest

The authors declare no conflicts of interest.

## Supporting Information

Additional supporting information can be found online in the Supporting Information section.

## Supporting information


**Supporting Information** Supporting Information associated with this article can be found in the online version. Figure S1 Results of the factor selection. (A) Plot of the LASSO coefficient profiles. (B) Tuning parameter (*λ*) selection cross‐validation error curve. Figure S2 Results of the factor selection. (A) Plot of the LASSO coefficient profiles. (B) Tuning parameter (*λ*) selection cross‐validation error curve. Figure S3 ROC for 7‐day intubation by different indicators. Figure S4 ROC for 28‐day mortality by different indicators. Figure S5: Kaplan–Meier survival curves showing intubation rates within 7 days across the three SR groups (HRR). Figure S6: Kaplan–Meier survival curves showing 28‐day mortality across the three SR groups(HRR). Figure S7: Dose–response association between SR and 7‐day intubation and 28‐day mortality based on restricted cubic spline analyses. Models (B/D) were adjusted for sex, age, race, and BMI(HRR). Figure S8: Results of the factor selection. (A) Plot of the LASSO coefficient profiles. (B) Tuning parameter (*λ*) selection cross‐validation error curve. Figure S9: Results of the factor selection. (A) Plot of the LASSO coefficient profiles. (B) Tuning parameter (*λ*) selection cross‐validation error curve. Table S1: Univariate COX regression for7‐day intubation. Table S2:Univariate COX regression for 28‐day mortality. Table S3 Diagnostic values for 7‐day intubation by different indicators. Table S4 diagnostic values for 28‐day mortality by different indicators. Table S5: Baseline characteristics of patients(HRR). Table S6: The association between the SR and 7‐day intubation(HRR COX regression). Table S7: Univariate cox regression for 7‐day intubation(HRR). Table S8: The association between the SR and 28‐day mortality(HRR COX regression). Table S9: Univariate cox regression for 28‐day mortality(HRR). Table S10. Baseline characteristics of patients. Table S11. Baseline characteristics of patients. Table S12. Association between SR and 7‐day intubation. Table S13. Univariate COX regression for7‐day intubation. Table S14. Association between SR and 28‐day mortality. Table S15. Univariate COX regression for 28‐day mortality.

## Data Availability

The datasets used and/or analyzed during the current study are available from the corresponding author upon reasonable request.
