# Supplementary material for: Predictive Value of SpO2/RR Ratio for Short‐Term Intubation and Mortality Risks in Pre‐ED Patients With Acute Dyspnea Without Oxygen Therapy: A Retrospective Cohort Study
Source: Emerg Med Int. 2026 Jun 30;2026:1372222. doi: 10.1155/emmi/1372222 (PMC13318678; doi:10.1155/emmi/1372222)
Supplement: Supplementary file 1 — Supporting Information Supporting Information associated with this article can be found in the online version. Figure S1 Results of the factor selection. (A) Plot of the LASSO coefficient profiles. (B) Tuning parameter (λ) selection cross‐validation error curve. Figure S2 Results of the factor selection. (A) Plot of the LASSO coefficient profiles. (B) Tuning parameter (λ) selection cross‐validation error curve. Figure S3 ROC for 7‐day intubation by different indicators. Figure S4 ROC for 28‐day mortality by different indicators. Figure S5: Kaplan–Meier survival curves showing intubation rates within 7 days across the three SR groups (HRR). Figure S6: Kaplan–Meier survival curves showing 28‐day mortality across the three SR groups(HRR). Figure S7: Dose–response association between SR and 7‐day intubation and 28‐day mortality based on restricted cubic spline analyses. Models (B/D) were adjusted for sex, age, race, and BMI(HRR). Figure S8: Results of the factor selection. (A) Plot of the LASSO coefficient profiles. (B) Tuning parameter (λ) selection cross‐validation error curve. Figure S9: Results of the factor selection. (A) Plot of the LASSO coefficient profiles. (B) Tuning parameter (λ) selection cross‐validation error curve. Table S1: Univariate COX regression for7‐day intubation. Table S2:Univariate COX regression for 28‐day mortality. Table S3 Diagnostic values for 7‐day intubation by different indicators. Table S4 diagnostic values for 28‐day mortality by different indicators. Table S5: Baseline characteristics of patients(HRR). Table S6: The association between the SR and 7‐day intubation(HRR COX regression). Table S7: Univariate cox regression for 7‐day intubation(HRR). Table S8: The association between the SR and 28‐day mortality(HRR COX regression). Table S9: Univariate cox regression for 28‐day mortality(HRR). Table S10. Baseline characteristics of patients. Table S11. Baseline characteristics of patients. Table S12. Association between SR and 7‐day intubat [file EMMI-2026-1372222-s001.docx]

Table S1.Univariate COX regression for7-day intubation

| Variables | β | S.E | Z | *P* | HR (95%CI) |
| --- | --- | --- | --- | --- | --- |
|  |  |  |  |  |  |
| Age | 0.00 | 0.00 | 0.48 | 0.634 | 1.00 (0.99 ~ 1.01) |
| Sex |  |  |  |  |  |
| Male |  |  |  |  | 1.00 (Reference) |
| Female | -0.32 | 0.14 | -2.34 | **0.019** | 0.73 (0.56 ~ 0.95) |
| Race |  |  |  |  |  |
| Han ethnic group |  |  |  |  | 1.00 (Reference) |
| National minority | -0.17 | 0.71 | -0.23 | 0.815 | 0.85 (0.21 ~ 3.41) |
| UNKNOWN | 0.44 | 0.18 | 2.47 | **0.013** | 1.56 (1.10 ~ 2.21) |
| Hypertensive |  |  |  |  |  |
| No |  |  |  |  | 1.00 (Reference) |
| YES | 0.12 | 0.13 | 0.93 | 0.353 | 1.13 (0.87 ~ 1.46) |
| Diabetes |  |  |  |  |  |
| No |  |  |  |  | 1.00 (Reference) |
| YES | 0.44 | 0.13 | 3.31 | **<.001** | 1.55 (1.20 ~ 2.02) |
| Coronary heart disease |  |  |  |  |  |
| No |  |  |  |  | 1.00 (Reference) |
| YES | -0.15 | 0.20 | -0.74 | 0.459 | 0.86 (0.59 ~ 1.27) |
| Cerebral infarction |  |  |  |  |  |
| No |  |  |  |  | 1.00 (Reference) |
| YES | -0.39 | 0.17 | -2.28 | **0.023** | 0.68 (0.48 ~ 0.95) |
| Atrial fibrillation |  |  |  |  |  |
| No |  |  |  |  | 1.00 (Reference) |
| YES | -0.78 | 0.45 | -1.72 | 0.085 | 0.46 (0.19 ~ 1.11) |
| Cardiac insufficiency |  |  |  |  |  |
| No |  |  |  |  | 1.00 (Reference) |
| YES | -0.50 | 0.45 | -1.11 | 0.267 | 0.61 (0.25 ~ 1.47) |
| Alzheimers disease |  |  |  |  |  |
| No |  |  |  |  | 1.00 (Reference) |
| YES | 0.45 | 0.25 | 1.83 | 0.068 | 1.56 (0.97 ~ 2.53) |
| Bronchitis |  |  |  |  |  |
| No |  |  |  |  | 1.00 (Reference) |
| YES | -0.24 | 0.41 | -0.58 | 0.562 | 0.79 (0.35 ~ 1.77) |
| COPD |  |  |  |  |  |
| No |  |  |  |  | 1.00 (Reference) |
| YES | 0.13 | 0.30 | 0.45 | 0.652 | 1.14 (0.64 ~ 2.04) |
| Cirrhosis |  |  |  |  |  |
| No |  |  |  |  | 1.00 (Reference) |
| YES | -0.06 | 0.38 | -0.16 | 0.873 | 0.94 (0.44 ~ 2.00) |
| Renal insufficiency |  |  |  |  |  |
| No |  |  |  |  | 1.00 (Reference) |
| YES | 0.22 | 0.27 | 0.83 | 0.404 | 1.25 (0.74 ~ 2.11) |
| Malignant tumour |  |  |  |  |  |
| No |  |  |  |  | 1.00 (Reference) |
| YES | 0.21 | 0.19 | 1.11 | 0.265 | 1.24 (0.85 ~ 1.81) |
| Heart rate | 0.01 | 0.00 | 3.75 | **<.001** | 1.01 (1.01 ~ 1.01) |
| SBP | -0.00 | 0.00 | -0.47 | 0.635 | 1.00 (1.00 ~ 1.00) |
| DBP | -0.00 | 0.00 | -1.03 | 0.304 | 1.00 (0.99 ~ 1.00) |
| MBP | -0.00 | 0.00 | -0.90 | 0.369 | 1.00 (0.99 ~ 1.00) |
| Temperature | -0.80 | 0.58 | -1.38 | 0.167 | 0.45 (0.15 ~ 1.40) |
| PH | 0.02 | 0.08 | 0.22 | 0.829 | 1.02 (0.87 ~ 1.19) |
| PaO_2_ | -0.00 | 0.00 | -0.75 | 0.452 | 1.00 (1.00 ~ 1.00) |
| PaCO_2_ | 0.01 | 0.00 | 1.49 | 0.137 | 1.01 (1.00 ~ 1.01) |
| Lac | 0.04 | 0.02 | 2.39 | **0.017** | 1.04 (1.01 ~ 1.08) |
| Hscrp | 0.01 | 0.00 | 2.46 | **0.014** | 1.01 (1.01 ~ 1.01) |
| WBC | 0.00 | 0.01 | 0.44 | 0.662 | 1.00 (0.99 ~ 1.02) |
| PCT | 0.01 | 0.01 | 1.82 | 0.069 | 1.01 (1.00 ~ 1.02) |
| Tbil | 0.00 | 0.00 | 0.54 | 0.587 | 1.00 (1.00 ~ 1.00) |
| ALT | 0.00 | 0.00 | 0.95 | 0.342 | 1.00 (1.00 ~ 1.00) |
| AST | 0.00 | 0.00 | 1.67 | 0.095 | 1.00 (1.00 ~ 1.00) |
| Cr | 0.00 | 0.00 | 1.93 | 0.053 | 1.00 (1.00 ~ 1.00) |
| ALB | -0.00 | 0.01 | -0.08 | 0.935 | 1.00 (0.98 ~ 1.02) |
| BUN | 0.00 | 0.00 | 0.40 | 0.691 | 1.00 (0.99 ~ 1.01) |
| HB | 0.00 | 0.00 | 1.52 | 0.129 | 1.00 (1.00 ~ 1.01) |
| PLT | 0.00 | 0.00 | 0.18 | 0.861 | 1.00 (1.00 ~ 1.00) |
| PT | -0.00 | 0.02 | -0.17 | 0.864 | 1.00 (0.97 ~ 1.03) |
| APTT | 0.01 | 0.00 | 1.46 | 0.143 | 1.01 (1.00 ~ 1.02) |
| FIB | 0.04 | 0.02 | 2.26 | **0.024** | 1.04 (1.01 ~ 1.08) |
| DD | 0.00 | 0.00 | 0.81 | 0.417 | 1.00 (1.00 ~ 1.00) |

| 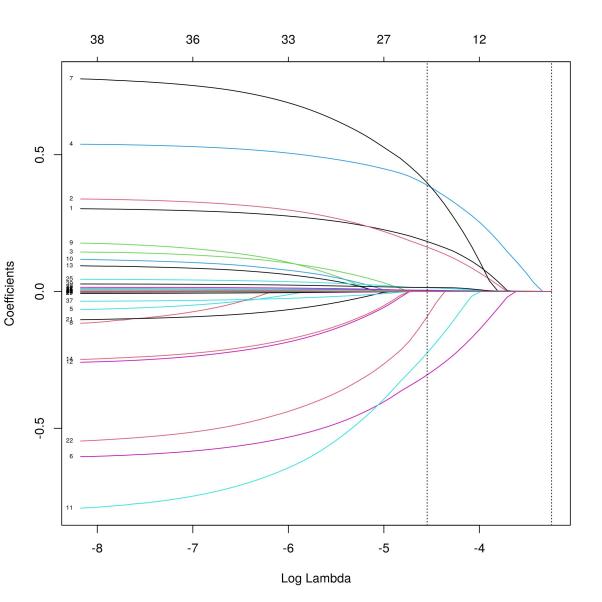  A | 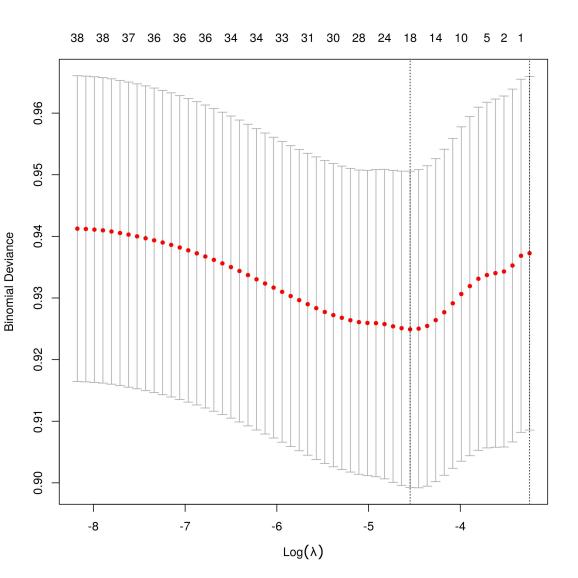  B |
| --- | --- |

Fig. S1 Results of the factor selection. A Plot of the LASSO coefficient profiles. B Tuning parameter (λ) selection cross-validation error curve.

Table S2.Univariate COX regression for 28-day mortality

| Variables | β | S.E | Z | *P* | HR (95%CI) |
| --- | --- | --- | --- | --- | --- |
|  |  |  |  |  |  |
| Age | 0.02 | 0.00 | 4.96 | **<.001** | 1.02 (1.01 ~ 1.03) |
| Sex |  |  |  |  |  |
| Male |  |  |  |  | 1.00 (Reference) |
| Female | -0.13 | 0.13 | -0.98 | 0.325 | 0.88 (0.67 ~ 1.14) |
| Race |  |  |  |  |  |
| Han ethnic group |  |  |  |  | 1.00 (Reference) |
| National minority | 0.31 | 0.58 | 0.54 | 0.590 | 1.37 (0.44 ~ 4.28) |
| UNKNOWN | 0.22 | 0.20 | 1.12 | 0.262 | 1.25 (0.85 ~ 1.83) |
| Hypertensive |  |  |  |  |  |
| No |  |  |  |  | 1.00 (Reference) |
| YES | 0.18 | 0.13 | 1.33 | 0.183 | 1.19 (0.92 ~ 1.55) |
| Diabetes |  |  |  |  |  |
| No |  |  |  |  | 1.00 (Reference) |
| YES | 0.09 | 0.14 | 0.62 | 0.535 | 1.09 (0.83 ~ 1.44) |
| Coronary heart disease |  |  |  |  |  |
| No |  |  |  |  | 1.00 (Reference) |
| YES | -0.19 | 0.20 | -0.92 | 0.358 | 0.83 (0.55 ~ 1.24) |
| Cerebral infarction |  |  |  |  |  |
| No |  |  |  |  | 1.00 (Reference) |
| YES | 0.31 | 0.15 | 2.15 | **0.031** | 1.37 (1.03 ~ 1.82) |
| Atrial fibrillation |  |  |  |  |  |
| No |  |  |  |  | 1.00 (Reference) |
| YES | 0.09 | 0.31 | 0.30 | 0.764 | 1.10 (0.60 ~ 2.01) |
| Cardiac insufficiency |  |  |  |  |  |
| No |  |  |  |  | 1.00 (Reference) |
| YES | 0.44 | 0.31 | 1.41 | 0.159 | 1.55 (0.84 ~ 2.83) |
| Alzheimers disease |  |  |  |  |  |
| No |  |  |  |  | 1.00 (Reference) |
| YES | 0.06 | 0.30 | 0.21 | 0.835 | 1.06 (0.59 ~ 1.90) |
| Bronchitis |  |  |  |  |  |
| No |  |  |  |  | 1.00 (Reference) |
| YES | 0.37 | 0.32 | 1.13 | 0.259 | 1.44 (0.76 ~ 2.71) |
| COPD |  |  |  |  |  |
| No |  |  |  |  | 1.00 (Reference) |
| YES | 0.11 | 0.31 | 0.37 | 0.711 | 1.12 (0.61 ~ 2.05) |
| Cirrhosis |  |  |  |  |  |
| No |  |  |  |  | 1.00 (Reference) |
| YES | 0.53 | 0.31 | 1.71 | 0.087 | 1.70 (0.93 ~ 3.11) |
| Renal insufficiency |  |  |  |  |  |
| No |  |  |  |  | 1.00 (Reference) |
| YES | -0.19 | 0.32 | -0.57 | 0.565 | 0.83 (0.44 ~ 1.57) |
| Malignant tumour |  |  |  |  |  |
| No |  |  |  |  | 1.00 (Reference) |
| YES | 0.92 | 0.16 | 5.73 | **<.001** | 2.50 (1.83 ~ 3.42) |
| Heart rate | 0.00 | 0.00 | 1.57 | 0.116 | 1.00 (1.00 ~ 1.00) |
| SBP | -0.01 | 0.00 | -5.56 | **<.001** | 0.99 (0.98 ~ 0.99) |
| DBP | -0.02 | 0.00 | -4.63 | **<.001** | 0.98 (0.98 ~ 0.99) |
| MBP | -0.02 | 0.00 | -5.40 | **<.001** | 0.98 (0.98 ~ 0.99) |
| Temperature | -1.08 | 0.56 | -1.93 | 0.054 | 0.34 (0.11 ~ 1.02) |
| PH | -0.05 | 0.09 | -0.61 | 0.545 | 0.95 (0.80 ~ 1.12) |
| PaO_2_ | 0.00 | 0.00 | 1.01 | 0.311 | 1.00 (1.00 ~ 1.00) |
| PaCO_2_ | -0.01 | 0.01 | -2.17 | **0.030** | 0.99 (0.98 ~ 0.99) |
| Lac | 0.09 | 0.01 | 6.15 | **<.001** | 1.09 (1.06 ~ 1.12) |
| Hscrp | 0.01 | 0.00 | 7.10 | **<.001** | 1.01 (1.01 ~ 1.01) |
| WBC | 0.02 | 0.00 | 4.95 | **<.001** | 1.02 (1.01 ~ 1.03) |
| PCT | 0.02 | 0.00 | 4.66 | **<.001** | 1.02 (1.01 ~ 1.03) |
| Tbil | 0.01 | 0.00 | 4.47 | **<.001** | 1.01 (1.01 ~ 1.01) |
| ALT | 0.01 | 0.00 | 3.29 | **<.001** | 1.01 (1.01 ~ 1.01) |
| AST | 0.01 | 0.00 | 2.28 | **0.023** | 1.01 (1.01 ~ 1.01) |
| Cr | 0.01 | 0.00 | 4.34 | **<.001** | 1.01 (1.01 ~ 1.01) |
| ALB | -0.07 | 0.01 | -7.61 | **<.001** | 0.93 (0.92 ~ 0.95) |
| BUN | 0.01 | 0.00 | 6.02 | **<.001** | 1.01 (1.01 ~ 1.01) |
| HB | -0.01 | 0.00 | -6.22 | **<.001** | 0.99 (0.98 ~ 0.99) |
| PLT | 0.00 | 0.00 | 0.53 | 0.597 | 1.00 (1.00 ~ 1.00) |
| PT | 0.03 | 0.01 | 4.68 | **<.001** | 1.03 (1.02 ~ 1.04) |
| APTT | 0.01 | 0.00 | 2.42 | **0.016** | 1.01 (1.01 ~ 1.02) |
| FIB | 0.03 | 0.02 | 1.17 | 0.242 | 1.03 (0.98 ~ 1.07) |
| DD | -0.00 | 0.00 | -1.60 | 0.111 | 1.00 (1.00 ~ 1.00) |

| 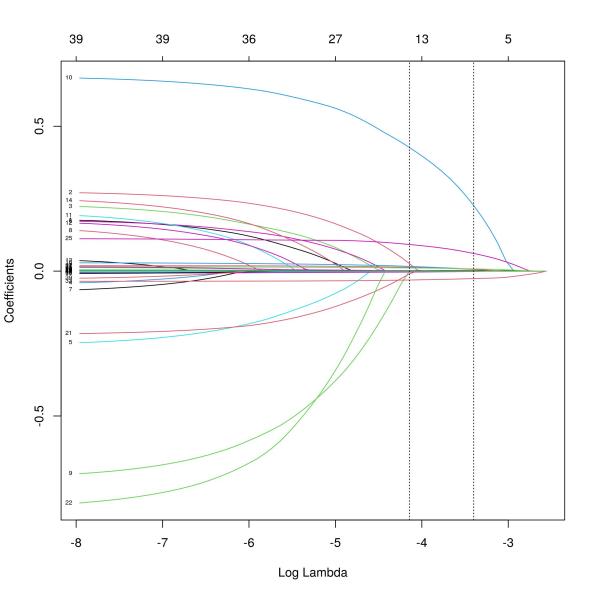  A | 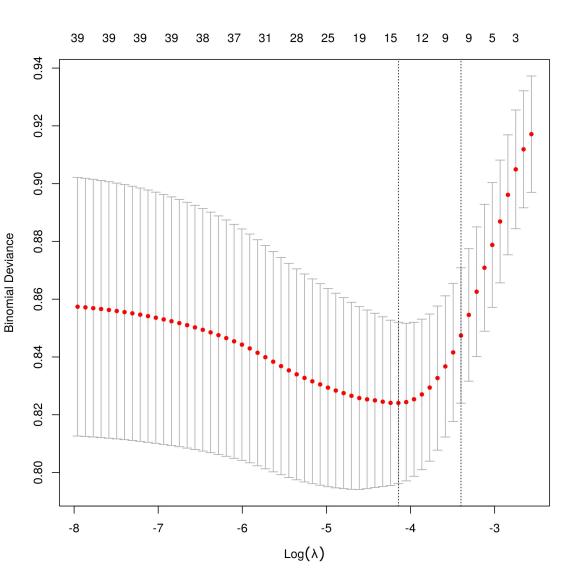  B |
| --- | --- |

Fig. S2 Results of the factor selection. A Plot of the LASSO coefficient profiles. B Tuning parameter (λ) selection cross-validation error curve.

Table S3 diagnostic values for 7-day intubation by different indicators

| Features | AUC | Sensitivity | Specificity | Jorden Index | Optimal threshold | Accuracy |
| --- | --- | --- | --- | --- | --- | --- |
| SR | 0.564(0.516-0.607) | 0.659(0.564-0.871) | 0.479(0.235-0.580) | 0.138(0.083-0.209) | 4.364(3.393-4.700) | 0.649(0.563-0.764) |
| RR | 0.538(0.494-0.582) | 0.471(0.170-0.527) | 0.630(0.612-0.913) | 0.101(0.057-0.173) | 21.000(21.000-30.000) | 0.648(0.586-0.784) |
| SpO_2_ | 0.599(0.556-0.641) | 0.575(0.484-0.842) | 0.601(0.319-0.701) | 0.176(0.119-0.253) | 96.000(88.000-97.000) | 0.619(0.522-0.748) |
| age | 0.503(0.464-0.541) | 0.752(0.390-0.975) | 0.299(0.053-0.662) | 0.051(0.017-0.113) | 65.000(37.000-80.000) | 0.357(0.218-0.618) |
| PH | 0.510(0.466-0.558) | 0.802(0.614-0.864) | 0.286(0.208-0.484) | 0.088(0.037-0.159) | 7.346(7.314-7.387) | 0.690(0.591-0.752) |
| PaO_2_ | 0.530(0.489-0.572) | 0.657(0.229-0.808) | 0.420(0.273-0.830) | 0.077(0.037-0.156) | 78.600(65.700-119.030) | 0.585(0.337-0.713) |
| PaCO_2_ | 0.517(0.473-0.557) | 0.336(0.067-0.755) | 0.735(0.296-0.978) | 0.071(0.031-0.148) | 41.210(31.995-68.800) | 0.662(0.380-0.815) |
| Lac | 0.557(0.516-0.597) | 0.588(0.173-0.887) | 0.501(0.210-0.919) | 0.090(0.062-0.170) | 2.106(1.210-5.789) | 0.594(0.326-0.791) |
| hsCRP | 0.578(0.540-0.611) | 0.693(0.434-0.885) | 0.440(0.263-0.699) | 0.133(0.099-0.205) | 4.897(0.990-32.780) | 0.503(0.368-0.655) |
| WBC | 0.554(0.512-0.595) | 0.445(0.405-0.861) | 0.670(0.256-0.709) | 0.116(0.074-0.193) | 11.150(6.610-11.550) | 0.522(0.363-0.663) |
| PCT | 0.536(0.495-0.578) | 0.408(0.220-0.884) | 0.674(0.190-0.852) | 0.081(0.044-0.160) | 0.362(0.047-1.646) | 0.584(0.316-0.744) |


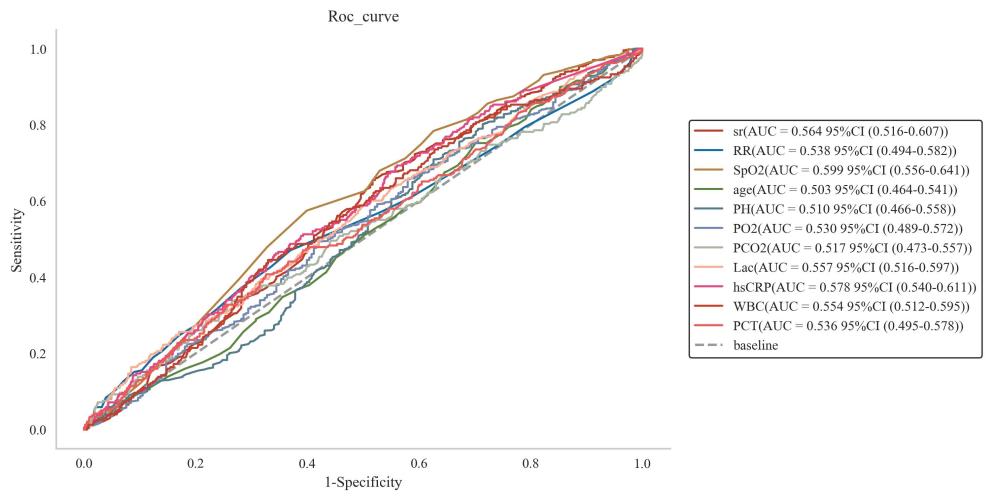


Fig S3 ROC for 7-day intubation by different indicators

Table S4 diagnostic values for 28-day mortality by different indicators

| Features | AUC | Sensitivity | Specificity | Jorden Index | Optimal threshold | Accuracy |
| --- | --- | --- | --- | --- | --- | --- |
| SR | 0.624(0.586-0.661) | 0.537(0.485-0.938) | 0.652(0.240-0.732) | 0.190(0.151-0.267) | 4.789(2.920-4.950) | 0.666(0.524-0.822) |
| RR | 0.604(0.565-0.645) | 0.291(0.251-0.699) | 0.877(0.478-0.895) | 0.169(0.123-0.243) | 27.000(20.000-27.000) | 0.715(0.515-0.802) |
| SpO_2_ | 0.605(0.560-0.651) | 0.720(0.584-0.868) | 0.470(0.306-0.611) | 0.190(0.135-0.266) | 93.000(88.000-96.000) | 0.691(0.590-0.776) |
| age | 0.605(0.567-0.642) | 0.622(0.542-0.924) | 0.549(0.236-0.607) | 0.171(0.130-0.246) | 75.000(60.000-77.575) | 0.505(0.349-0.603) |
| PH | 0.516(0.475-0.559) | 0.805(0.608-0.946) | 0.274(0.120-0.486) | 0.079(0.037-0.148) | 7.344(7.250-7.388) | 0.696(0.585-0.794) |
| PaO_2_ | 0.513(0.470-0.554) | 0.957(0.228-0.977) | 0.105(0.096-0.836) | 0.061(0.038-0.131) | 50.600(50.600-131.000) | 0.488(0.240-0.738) |
| PaCO_2_ | 0.550(0.504-0.592) | 0.766(0.581-0.938) | 0.357(0.182-0.574) | 0.123(0.084-0.203) | 31.440(23.700-35.260) | 0.706(0.576-0.806) |
| Lac | 0.609(0.566-0.648) | 0.430(0.262-0.684) | 0.758(0.519-0.907) | 0.189(0.136-0.253) | 3.300(2.090-5.200) | 0.708(0.543-0.794) |
| hsCRP | 0.670(0.632-0.707) | 0.648(0.502-0.789) | 0.637(0.485-0.772) | 0.284(0.231-0.356) | 17.370(5.870-40.970) | 0.642(0.536-0.731) |
| WBC | 0.615(0.569-0.651) | 0.509(0.419-0.740) | 0.708(0.482-0.777) | 0.217(0.149-0.283) | 11.510(8.810-12.454) | 0.667(0.524-0.721) |
| PCT | 0.614(0.573-0.656) | 0.578(0.322-0.645) | 0.610(0.590-0.854) | 0.189(0.138-0.273) | 0.223(0.223-1.571) | 0.653(0.591-0.765) |


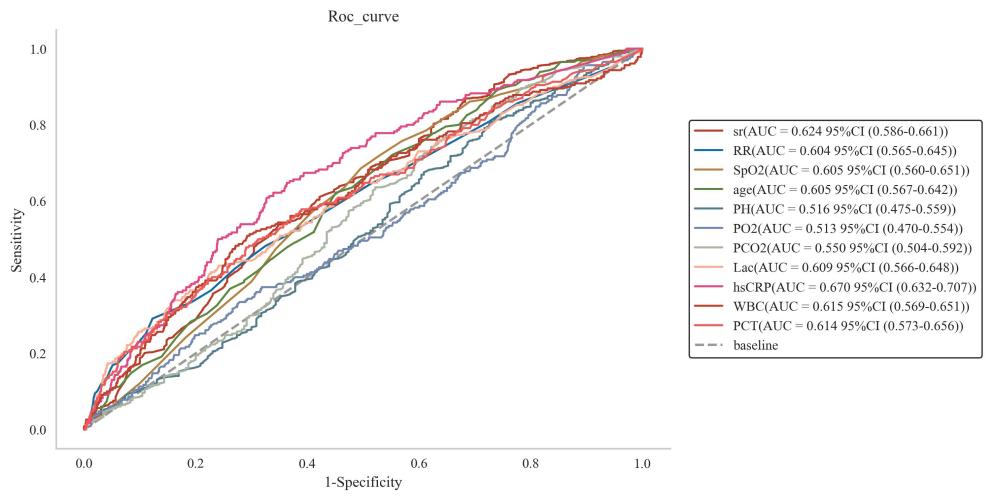


Fig S4 ROC for 28-day mortality by different indicators

Table S5 Baseline characteristics of patients（HRR）

| Variables | G1 (n = 45) | G2 (n = 45) | G3 (n = 45) | Statistic | *P* |
| --- | --- | --- | --- | --- | --- |
|  |  |  |  |  |  |
| SR | 2.04 ± 0.34 | 2.60 ± 0.11 | 3.04 ± 0.15 | F=222.64 | **<.001** |
| SPO_2_, % | 77.91 ± 14.62 | 90.69 ± 7.40 | 94.71 ± 4.13 | F=36.39 | **<.001** |
| RR, times/min | 38.24 ± 3.58 | 34.91 ± 3.17 | 31.22 ± 1.57 | F=65.81 | **<.001** |
| Age, years | 72.58 ± 15.13 | 75.74 ± 14.40 | 68.53 ± 14.89 | F=2.68 | 0.073 |
| BMI | 21.91 ± 4.80 | 23.12 ± 4.46 | 22.69 ± 4.56 | F=0.79 | 0.456 |
| Sex, n(%) |  |  |  | χ²=0.42 | 0.809 |
| Male | 27 (60.00) | 24 (53.33) | 26 (57.78) |  |  |
| Female | 18 (40.00) | 21 (46.67) | 19 (42.22) |  |  |
| Race, n(%) |  |  |  | - | 0.814 |
| Han ethnic group | 42 (93.33) | 40 (88.89) | 40 (88.89) |  |  |
| National minority | 0 (0.00) | 1 (2.22) | 0 (0.00) |  |  |
| UNKNOWN | 3 (6.67) | 4 (8.89) | 5 (11.11) |  |  |
| vital signs |  |  |  |  |  |
| Temperature, ℃ | 36.86 ± 0.73 | 36.87 ± 1.07 | 36.97 ± 1.21 | F=0.14 | 0.867 |
| Heart rate, times/min | 109.93 ± 24.45 | 105.91 ± 24.17 | 104.22 ± 25.40 | F=0.64 | 0.531 |
| SBP, mmhg | 136.29 ± 33.04 | 126.73 ± 29.78 | 137.29 ± 38.23 | F=1.33 | 0.267 |
| DBP, mmhg | 82.77 ± 23.20 | 76.29 ± 20.26 | 81.78 ± 23.62 | F=1.09 | 0.338 |
| MBP, mmhg | 100.61 ± 25.57 | 93.10 ± 22.43 | 100.28 ± 27.09 | F=1.29 | 0.280 |
| Laboratory values on admission |  |  |  |  |  |
| PH | 7.40 ± 0.11 | 7.35 ± 0.16 | 7.39 ± 0.09 | F=2.49 | 0.087 |
| PaO_2_, mmhg | 101.86 ± 38.79 | 92.87 ± 38.21 | 96.79 ± 33.21 | F=0.67 | 0.512 |
| PaCO_2_, mmhg | 42.09 ± 13.89 | 38.48 ± 16.79 | 37.53 ± 12.86 | F=1.22 | 0.297 |
| Lac, mmol/l | 2.70 (1.50,5.86) | 3.40 (1.85,4.96) | 2.40 (1.80,4.74) | χ²=0.39# | 0.823 |
| hsCRP, mg/L | 32.78 (6.16,113.70) | 45.64 (5.36,153.17) | 23.08 (6.74,65.00) | χ²=1.69# | 0.429 |
| WBC, ×10^9^ | 12.37 (8.24,16.00) | 11.80 (8.22,15.79) | 10.82 (8.42,12.93) | χ²=1.75# | 0.417 |
| PCT, ng/ml | 0.28 (0.07,1.60) | 0.26 (0.07,2.12) | 0.21 (0.07,1.69) | χ²=0.01# | 0.995 |
| Tbil,umol/l | 15.10 (12.30,24.30) | 19.95 (12.60,31.00) | 21.20 (13.10,30.20) | χ²=2.58# | 0.275 |
| ALT,U/L | 29.00 (18.00,47.00) | 27.80 (22.00,55.00) | 31.00 (22.00,64.80) | χ²=1.20# | 0.548 |
| AST,U/L | 30.00 (23.00,45.00) | 29.00 (23.00,56.00) | 40.00 (22.00,62.00) | χ²=1.32# | 0.517 |
| Creatinine, mg/dL | 88.80 (58.20,145.30) | 109.20 (73.30,163.60) | 77.50 (56.70,147.30) | χ²=3.29# | 0.193 |
| ALB, g/L | 34.00 (31.80,39.70) | 34.20 (28.90,40.30) | 35.90 (30.50,39.00) | χ²=0.29# | 0.866 |
| BUN,mg/dL | 8.20 (6.00,13.00) | 9.40 (6.80,15.30) | 8.03 (6.00,12.60) | χ²=2.25# | 0.324 |
| Hb, g/L | 123.00 (98.00,133.00) | 121.00 (107.00,137.00) | 131.00 (101.00,143.00) | χ²=0.87# | 0.647 |
| PLT, ×10^9^ | 199.40 (148.00,278.00) | 211.00 (158.00,276.00) | 181.00 (132.00,247.00) | χ²=1.87# | 0.393 |
| PT,s | 14.90 (14.30,16.00) | 15.10 (14.10,16.90) | 14.70 (13.70,16.30) | χ²=1.26# | 0.533 |
| APTT, s | 36.10 (33.60,41.00) | 36.60 (32.30,42.20) | 34.80 (32.30,38.50) | χ²=2.68# | 0.262 |
| FIB, g/L | 3.99 (3.31,5.70) | 4.41 (3.62,5.74) | 3.94 (3.23,4.96) | χ²=1.97# | 0.374 |
| DD, mg/L | 6.39 (1.79,76.16) | 5.23 (2.20,114.48) | 2.21 (0.87,85.02) | χ²=2.54# | 0.281 |
| Comorbidites, *n* (%) |  |  |  |  |  |
| Hypertensive |  |  |  | χ²=3.78 | 0.151 |
| No | 19 (42.22) | 16 (35.56) | 25 (55.56) |  |  |
| Yes | 26 (57.78) | 29 (64.44) | 20 (44.44) |  |  |
| Diabetes |  |  |  | χ²=1.40 | 0.497 |
| No | 27 (60.00) | 31 (68.89) | 32 (71.11) |  |  |
| Yes | 18 (40.00) | 14 (31.11) | 13 (28.89) |  |  |
| Coronary heart disease |  |  |  | χ²=3.46 | 0.177 |
| No | 39 (86.67) | 42 (93.33) | 36 (80.00) |  |  |
| Yes | 6 (13.33) | 3 (6.67) | 9 (20.00) |  |  |
| Cardiac insufficiency |  |  |  | - | 1.000 |
| No | 43 (95.56) | 43 (95.56) | 42 (93.33) |  |  |
| Yes | 2 (4.44) | 2 (4.44) | 3 (6.67) |  |  |
| Atrial fibrillation |  |  |  | - | 0.909 |
| No | 43 (95.56) | 41 (91.11) | 42 (93.33) |  |  |
| Yes | 2 (4.44) | 4 (8.89) | 3 (6.67) |  |  |
| Cerebral infarction |  |  |  | χ²=0.54 | 0.763 |
| No | 32 (71.11) | 33 (73.33) | 35 (77.78) |  |  |
| Yes | 13 (28.89) | 12 (26.67) | 10 (22.22) |  |  |
| Alzheimers disease |  |  |  | - | 0.789 |
| No | 42 (93.33) | 40 (88.89) | 42 (93.33) |  |  |
| Yes | 3 (6.67) | 5 (11.11) | 3 (6.67) |  |  |
| Bronchitis |  |  |  | - | 0.593 |
| No | 39 (86.67) | 42 (93.33) | 42 (93.33) |  |  |
| Yes | 6 (13.33) | 3 (6.67) | 3 (6.67) |  |  |
| COPD |  |  |  | - | 1.000 |
| No | 41 (91.11) | 41 (91.11) | 41 (91.11) |  |  |
| Yes | 4 (8.89) | 4 (8.89) | 4 (8.89) |  |  |
| Cirrhosis |  |  |  | - | 0.370 |
| No | 45 (100.00) | 43 (95.56) | 42 (93.33) |  |  |
| Yes | 0 (0.00) | 2 (4.44) | 3 (6.67) |  |  |
| Renal insufficiency |  |  |  | - | 0.165 |
| No | 45 (100.00) | 41 (91.11) | 43 (95.56) |  |  |
| Yes | 0 (0.00) | 4 (8.89) | 2 (4.44) |  |  |
| Malignant tumour |  |  |  | χ²=2.72 | 0.257 |
| No | 36 (80.00) | 36 (80.00) | 41 (91.11) |  |  |
| Yes | 9 (20.00) | 9 (20.00) | 4 (8.89) |  |  |
| Outcome |  |  |  |  |  |
| 7day-Intubation |  |  |  | χ²=2.95 | 0.228 |
| No | 29 (64.44) | 34 (75.56) | 36 (80.00) |  |  |
| Yes | 16 (35.56) | 11 (24.44) | 9 (20.00) |  |  |
| 28-day Mortality |  |  |  | χ²=6.53 | **0.038** |
| No | 26 (57.78) | 26 (57.78) | 36 (80.00) |  |  |
| Yes | 19 (42.22) | 19 (42.22) | 9 (20.00) |  |  |


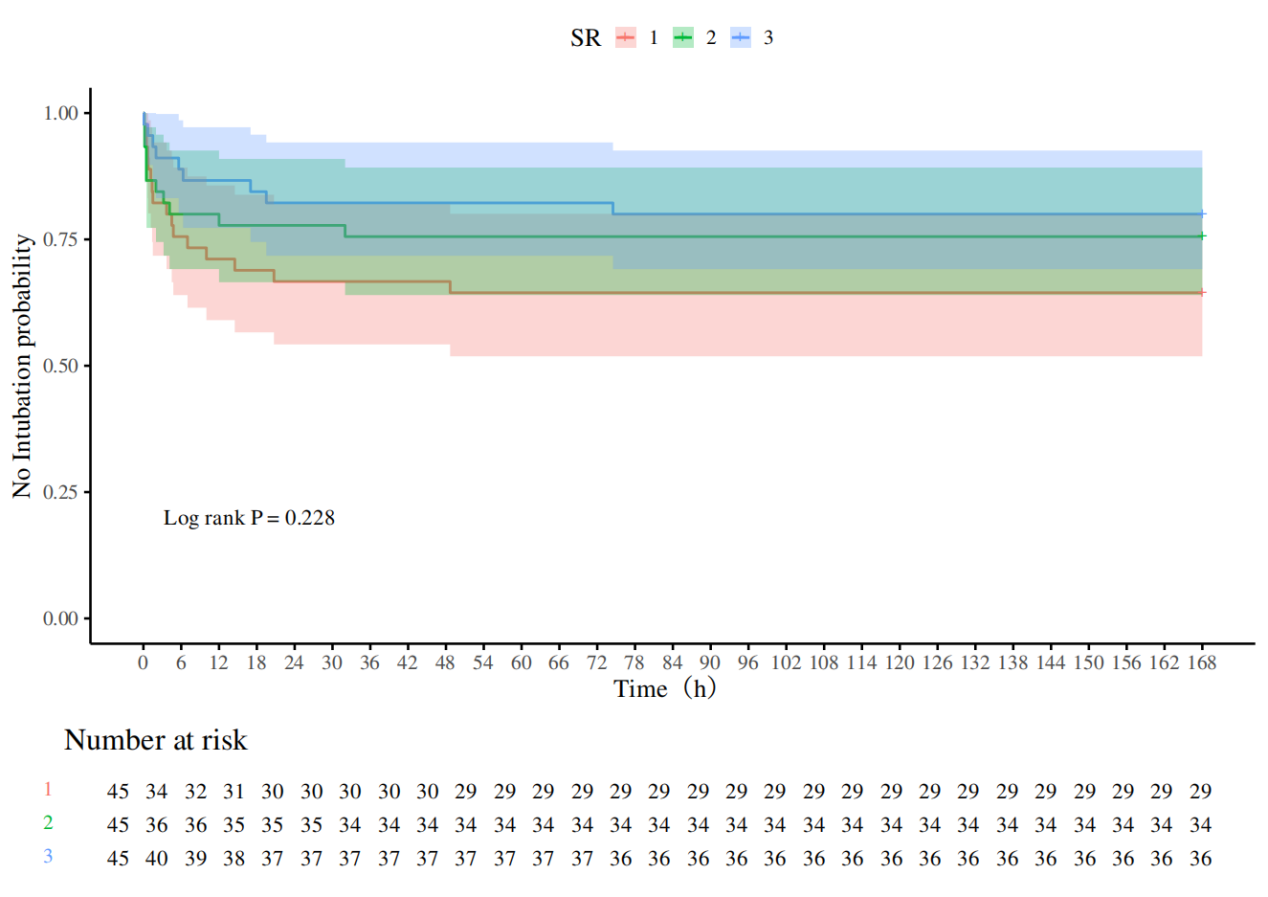


Fig. S5. Kaplan–Meier survival curves showing intubation rates within 7 days across the three SR groups


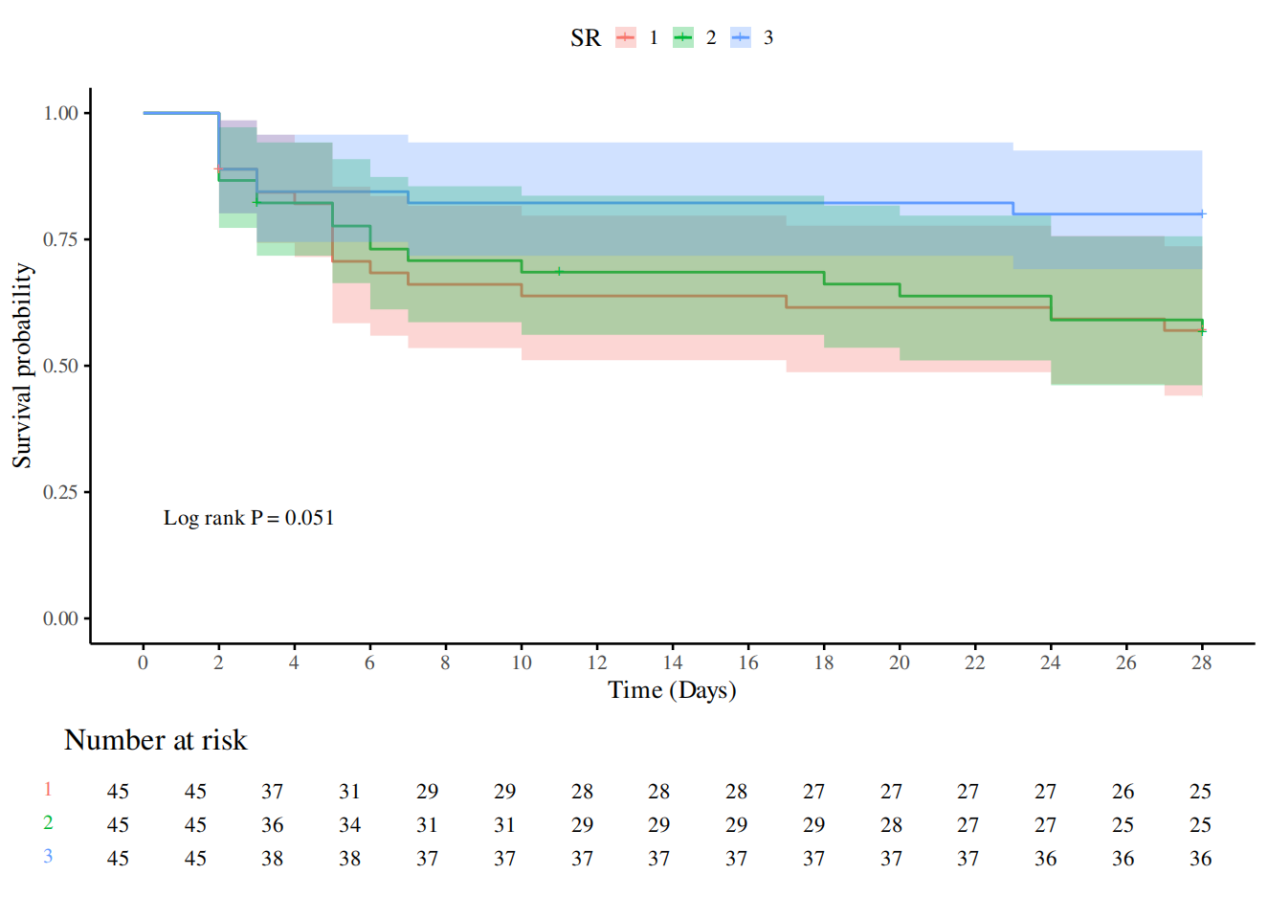


Fig. S6. Kaplan–Meier survival curves showing 28-day mortality across the three SR groups

Table S6 The association between the SR and 7-day intubation（HRR COX regression）

| Variables | Model1 | |  | Model2 | |  | Model3 | |
| --- | --- | --- | --- | --- | --- | --- | --- | --- |
|  | HR (95%CI) | *P* |  | HR (95%CI) | *P* |  | HR (95%CI) | *P* |
| SR | 0.50 (0.27 ~ 0.91) | **0.023** |  | 0.50 (0.27 ~ 0.92) | **0.025** |  | 0.49 (0.26 ~ 0.92) | **0.027** |
| SR group |  |  |  |  |  |  |  |  |
| 1 | 1.00 (Reference) |  |  | 1.00 (Reference) |  |  | 1.00 (Reference) |  |
| 2 | 0.67 (0.31 ~ 1.44) | 0.303 |  | 0.74 (0.34 ~ 1.61) | 0.445 |  | 0.52 (0.23 ~ 1.17) | 0.112 |
| 3 | 0.51 (0.22 ~ 1.15) | 0.103 |  | 0.49 (0.21 ~ 1.11) | 0.085 |  | 0.49 (0.21 ~ 1.12) | 0.089 |
| HR: Hazard Ratio, CI: Confidence Interval | | | | | | | | |
| Model1: Crude | | | | | | | | |
| Model2: Adjust: Sex,Race, BMI, Age | | | | | | | | |
| Model3: Adjust: Sex,Race, BMI, Age,Cerebral infarction | | | | | | | | |

Table S7 Univariate cox regression for 7-day intubation(HRR)

| Variables | β | S.E | Z | *P* | HR (95%CI) |
| --- | --- | --- | --- | --- | --- |
|  |  |  |  |  |  |
| Age | -0.01 | 0.01 | -0.67 | 0.504 | 0.99 (0.97 ~ 1.01) |
| Sex |  |  |  |  |  |
| Male |  |  |  |  | 1.00 (Reference) |
| Female | 0.11 | 0.34 | 0.33 | 0.739 | 1.12 (0.58 ~ 2.16) |
| Race |  |  |  |  |  |
| Han ethnic group |  |  |  |  | 1.00 (Reference) |
| National minority | -14.99 | 3352.04 | -0.00 | 0.996 | 0.00 (0.00 ~ Inf) |
| UNKNOWN | 0.74 | 0.48 | 1.54 | 0.124 | 2.10 (0.82 ~ 5.41) |
| Hypertensive |  |  |  |  |  |
| No |  |  |  |  | 1.00 (Reference) |
| Yes | -0.10 | 0.33 | -0.30 | 0.768 | 0.91 (0.47 ~ 1.74) |
| Diabetes |  |  |  |  |  |
| No |  |  |  |  | 1.00 (Reference) |
| Yes | 0.17 | 0.35 | 0.48 | 0.634 | 1.18 (0.60 ~ 2.33) |
| Coronary heart disease |  |  |  |  |  |
| No |  |  |  |  | 1.00 (Reference) |
| Yes | -0.28 | 0.53 | -0.54 | 0.592 | 0.75 (0.27 ~ 2.13) |
| Cerebral infarction |  |  |  |  |  |
| No |  |  |  |  | 1.00 (Reference) |
| Yes | -1.19 | 0.53 | -2.25 | **0.025** | 0.30 (0.11 ~ 0.86) |
| Atrial fibrillation |  |  |  |  |  |
| No |  |  |  |  | 1.00 (Reference) |
| Yes | -0.15 | 0.73 | -0.21 | 0.835 | 0.86 (0.21 ~ 3.58) |
| Cardiac insufficiency |  |  |  |  |  |
| No |  |  |  |  | 1.00 (Reference) |
| Yes | -0.77 | 1.01 | -0.76 | 0.447 | 0.46 (0.06 ~ 3.37) |
| Alzheimers disease |  |  |  |  |  |
| No |  |  |  |  | 1.00 (Reference) |
| Yes | 0.32 | 0.53 | 0.61 | 0.544 | 1.38 (0.49 ~ 3.90) |
| Bronchitis |  |  |  |  |  |
| No |  |  |  |  | 1.00 (Reference) |
| Yes | -0.59 | 0.73 | -0.82 | 0.414 | 0.55 (0.13 ~ 2.30) |
| COPD |  |  |  |  |  |
| No |  |  |  |  | 1.00 (Reference) |
| Yes | 0.22 | 0.53 | 0.42 | 0.673 | 1.25 (0.44 ~ 3.54) |
| Cirrhosis |  |  |  |  |  |
| No |  |  |  |  | 1.00 (Reference) |
| Yes | -17.07 | 4013.49 | -0.00 | 0.997 | 0.00 (0.00 ~ Inf) |
| Renal insufficiency |  |  |  |  |  |
| No |  |  |  |  | 1.00 (Reference) |
| Yes | -17.08 | 3673.66 | -0.00 | 0.996 | 0.00 (0.00 ~ Inf) |
| Malignant tumour |  |  |  |  |  |
| No |  |  |  |  | 1.00 (Reference) |
| Yes | 0.02 | 0.45 | 0.04 | 0.968 | 1.02 (0.42 ~ 2.45) |
| Heart rate | 0.00 | 0.01 | 0.70 | 0.481 | 1.00 (0.99 ~ 1.02) |
| SBP | -0.01 | 0.01 | -1.23 | 0.220 | 0.99 (0.98 ~ 1.00) |
| DBP | -0.01 | 0.01 | -1.27 | 0.204 | 0.99 (0.97 ~ 1.01) |
| MBP | -0.01 | 0.01 | -1.31 | 0.189 | 0.99 (0.98 ~ 1.00) |
| Temperature | -0.08 | 0.18 | -0.43 | 0.669 | 0.93 (0.65 ~ 1.31) |
| PH | -1.21 | 1.31 | -0.92 | 0.357 | 0.30 (0.02 ~ 3.91) |
| PaO_2_ | 0.01 | 0.00 | 1.38 | 0.169 | 1.01 (1.00 ~ 1.02) |
| PaCO_2_ | 0.00 | 0.01 | 0.29 | 0.768 | 1.00 (0.98 ~ 1.03) |
| Lac | 0.02 | 0.04 | 0.49 | 0.624 | 1.02 (0.94 ~ 1.12) |
| HsCRP | 0.00 | 0.00 | 0.32 | 0.749 | 1.00 (1.00 ~ 1.00) |
| WBC | 0.01 | 0.03 | 0.19 | 0.847 | 1.01 (0.95 ~ 1.06) |
| PCT | 0.01 | 0.01 | 0.67 | 0.505 | 1.01 (0.99 ~ 1.03) |
| Tbil | -0.00 | 0.01 | -0.46 | 0.649 | 1.00 (0.98 ~ 1.01) |
| ALT | -0.00 | 0.00 | -0.47 | 0.637 | 1.00 (1.00 ~ 1.00) |
| AST | -0.00 | 0.00 | -0.49 | 0.626 | 1.00 (1.00 ~ 1.00) |
| Cr | 0.00 | 0.00 | 0.57 | 0.572 | 1.00 (1.00 ~ 1.00) |
| ALB | 0.03 | 0.03 | 1.32 | 0.187 | 1.03 (0.98 ~ 1.09) |
| BUN | 0.00 | 0.02 | 0.09 | 0.930 | 1.00 (0.97 ~ 1.04) |
| HB | 0.00 | 0.01 | 0.57 | 0.566 | 1.00 (0.99 ~ 1.01) |
| PLT | -0.00 | 0.00 | -0.32 | 0.748 | 1.00 (1.00 ~ 1.00) |
| PT | -0.09 | 0.08 | -1.24 | 0.217 | 0.91 (0.78 ~ 1.06) |
| APTT | -0.01 | 0.02 | -0.44 | 0.661 | 0.99 (0.95 ~ 1.04) |
| FIB | 0.05 | 0.08 | 0.63 | 0.526 | 1.05 (0.90 ~ 1.24) |
| DD | 0.00 | 0.00 | 0.33 | 0.740 | 1.00 (1.00 ~ 1.00) |

Table S8 The association between the SR and 28-day mortality（HRR COX regression）

| Variables | Model1 | |  | Model2 | |  | Model3 | |
| --- | --- | --- | --- | --- | --- | --- | --- | --- |
|  | HR (95%CI) | *P* |  | HR (95%CI) | *P* |  | HR (95%CI) | *P* |
| SR | 0.50 (0.29 ~ 0.85) | **0.010** |  | 0.44 (0.24 ~ 0.78) | **0.005** |  | 0.42 (0.22 ~ 0.80) | **0.008** |
| SR group |  |  |  |  |  |  |  |  |
| 1 | 1.00 (Reference) |  |  | 1.00 (Reference) |  |  | 1.00 (Reference) |  |
| 2 | 0.99 (0.52 ~ 1.86) | 0.965 |  | 0.88 (0.46 ~ 1.69) | 0.698 |  | 0.75 (0.37 ~ 1.51) | 0.415 |
| 3 | 0.42 (0.19 ~ 0.94) | **0.034** |  | 0.41 (0.19 ~ 0.92) | **0.031** |  | 0.41 (0.18 ~ 0.97) | **0.041** |
| HR: Hazard Ratio, CI: Confidence Interval | | | | | | | | |
| Model1: Crude | | | | | | | | |
| Model2: Adjust: Sex,Race, BMI, Age | | | | | | | | |
| Model3: Adjust: Age, Malignant tumour, SBP, DBP, AST, ALB, BUN, PT | | | | | | | | |

Table S9 Univariate cox regression for 28-day mortality(HRR)

| Variables | β | S.E | Z | *P* | HR (95%CI) |
| --- | --- | --- | --- | --- | --- |
|  |  |  |  |  |  |
| Age | 0.02 | 0.01 | 1.75 | 0.081 | 1.02 (1.00 ~ 1.04) |
| Sex |  |  |  |  |  |
| Male |  |  |  |  | 1.00 (Reference) |
| Female | 0.22 | 0.29 | 0.75 | 0.453 | 1.25 (0.70 ~ 2.21) |
| Race |  |  |  |  |  |
| Han ethnic group |  |  |  |  | 1.00 (Reference) |
| National minority | 1.00 | 1.01 | 0.99 | 0.323 | 2.72 (0.37 ~ 19.82) |
| UNKNOWN | 0.70 | 0.44 | 1.59 | 0.111 | 2.01 (0.85 ~ 4.76) |
| Hypertensive |  |  |  |  |  |
| No |  |  |  |  | 1.00 (Reference) |
| Yes | 0.09 | 0.30 | 0.30 | 0.764 | 1.09 (0.61 ~ 1.95) |
| Diabetes |  |  |  |  |  |
| No |  |  |  |  | 1.00 (Reference) |
| Yes | 0.52 | 0.29 | 1.76 | 0.079 | 1.67 (0.94 ~ 2.98) |
| Coronary heart disease |  |  |  |  |  |
| No |  |  |  |  | 1.00 (Reference) |
| Yes | -0.36 | 0.47 | -0.77 | 0.443 | 0.70 (0.28 ~ 1.76) |
| Cerebral infarction |  |  |  |  |  |
| No |  |  |  |  | 1.00 (Reference) |
| Yes | 0.41 | 0.31 | 1.32 | 0.185 | 1.50 (0.82 ~ 2.75) |
| Atrial fibrillation |  |  |  |  |  |
| No |  |  |  |  | 1.00 (Reference) |
| Yes | -0.12 | 0.60 | -0.19 | 0.847 | 0.89 (0.28 ~ 2.87) |
| Cardiac insufficiency |  |  |  |  |  |
| No |  |  |  |  | 1.00 (Reference) |
| Yes | -0.24 | 0.72 | -0.34 | 0.738 | 0.78 (0.19 ~ 3.24) |
| Alzheimers disease |  |  |  |  |  |
| No |  |  |  |  | 1.00 (Reference) |
| Yes | -0.75 | 0.72 | -1.04 | 0.296 | 0.47 (0.11 ~ 1.94) |
| Bronchitis |  |  |  |  |  |
| No |  |  |  |  | 1.00 (Reference) |
| Yes | -0.48 | 0.60 | -0.80 | 0.423 | 0.62 (0.19 ~ 2.00) |
| COPD |  |  |  |  |  |
| No |  |  |  |  | 1.00 (Reference) |
| Yes | 0.22 | 0.47 | 0.47 | 0.639 | 1.25 (0.49 ~ 3.16) |
| Cirrhosis |  |  |  |  |  |
| No |  |  |  |  | 1.00 (Reference) |
| Yes | 0.96 | 0.60 | 1.61 | 0.108 | 2.62 (0.81 ~ 8.46) |
| Renal insufficiency |  |  |  |  |  |
| No |  |  |  |  | 1.00 (Reference) |
| Yes | -0.00 | 0.72 | -0.00 | 0.997 | 1.00 (0.24 ~ 4.11) |
| Malignant tumour |  |  |  |  |  |
| No |  |  |  |  | 1.00 (Reference) |
| Yes | 0.73 | 0.34 | 2.18 | **0.029** | 2.08 (1.08 ~ 4.01) |
| Heart rate | -0.00 | 0.01 | -0.84 | 0.399 | 1.00 (0.98 ~ 1.01) |
| SBP | -0.01 | 0.00 | -2.17 | **0.030** | 0.99 (0.98 ~ 0.99) |
| DBP | -0.02 | 0.01 | -2.38 | **0.017** | 0.98 (0.97 ~ 0.99) |
| MBP | -0.01 | 0.01 | -2.39 | **0.017** | 0.99 (0.97 ~ 0.99) |
| Temperature | -0.34 | 0.19 | -1.85 | 0.064 | 0.71 (0.49 ~ 1.02) |
| PH | -0.23 | 1.11 | -0.21 | 0.835 | 0.79 (0.09 ~ 7.05) |
| PaO_2_ | 0.01 | 0.00 | 1.64 | 0.101 | 1.01 (1.00 ~ 1.01) |
| PaCO_2_ | -0.01 | 0.01 | -0.93 | 0.353 | 0.99 (0.97 ~ 1.01) |
| Lac | 0.05 | 0.03 | 1.54 | 0.124 | 1.05 (0.99 ~ 1.13) |
| HsCRP | 0.00 | 0.00 | 1.69 | 0.091 | 1.00 (1.00 ~ 1.01) |
| WBC | 0.01 | 0.02 | 0.60 | 0.546 | 1.01 (0.97 ~ 1.06) |
| PCT | -0.00 | 0.01 | -0.18 | 0.855 | 1.00 (0.98 ~ 1.02) |
| Tbil | 0.01 | 0.01 | 1.62 | 0.105 | 1.01 (1.00 ~ 1.02) |
| ALT | 0.00 | 0.00 | 0.78 | 0.434 | 1.00 (1.00 ~ 1.00) |
| AST | 0.01 | 0.00 | 2.76 | **0.006** | 1.01 (1.01 ~ 1.01) |
| Cr | 0.00 | 0.00 | 0.94 | 0.349 | 1.00 (1.00 ~ 1.00) |
| ALB | -0.05 | 0.02 | -2.29 | **0.022** | 0.95 (0.91 ~ 0.99) |
| BUN | 0.03 | 0.01 | 2.67 | **0.008** | 1.03 (1.01 ~ 1.05) |
| HB | -0.01 | 0.00 | -1.48 | 0.138 | 0.99 (0.98 ~ 1.00) |
| PLT | 0.00 | 0.00 | 1.49 | 0.136 | 1.00 (1.00 ~ 1.00) |
| PT | 0.10 | 0.05 | 2.10 | **0.036** | 1.10 (1.01 ~ 1.20) |
| APTT | 0.00 | 0.02 | 0.19 | 0.851 | 1.00 (0.97 ~ 1.04) |
| FIB | -0.04 | 0.08 | -0.55 | 0.585 | 0.96 (0.82 ~ 1.12) |
| DD | -0.00 | 0.00 | -0.50 | 0.617 | 1.00 (1.00 ~ 1.00) |


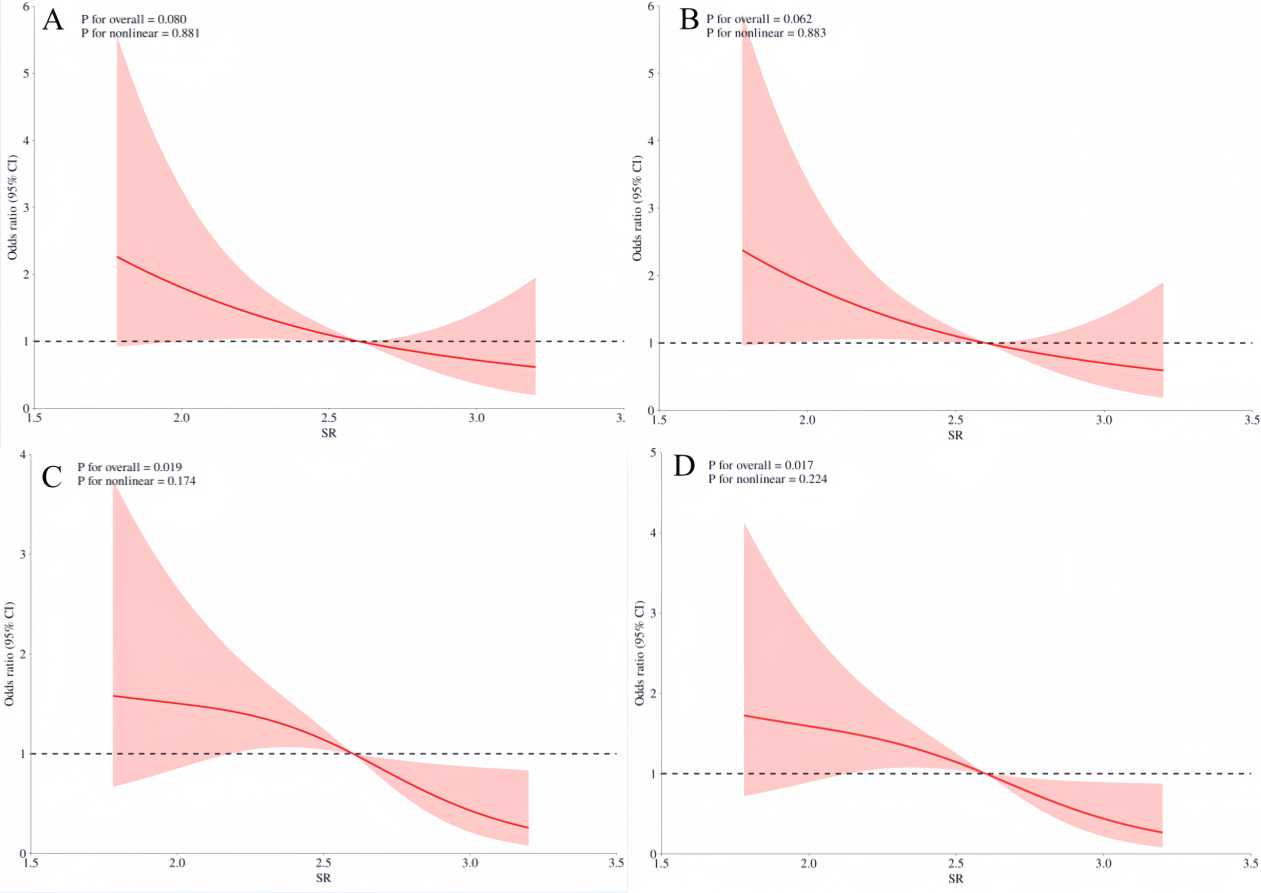


Fig. S7. Dose–response association between SR and 7-day intubation and 28-day mortality based on restricted cubic spline analyses. Models (B/D) were adjusted for sex, age, race, and body mass index (kg/m^2^). Reference values were set at the median SR. Solid lines represent adjusted odds ratios and hazard ratios, shaded areas represent 95% confidence intervals, and vertical dashed lines indicate the inflection points.

Table S10. Baseline characteristics of patients

| Variables | Exclusion (n = 726) | Included(n = 1339) | Statistic | *P* |
| --- | --- | --- | --- | --- |
|  |  |  |  |  |
| **Characteristic** |  |  |  |  |
| Age, years | 71.80 ± 15.90 | 70.81 ± 16.86 | t=1.31 | 0.191 |
| BMI | 22.55 ± 4.49 | 22.53 ± 4.58 | t=0.10 | 0.923 |
| Sex, *n(%)* |  |  | χ²=1.20 | 0.274 |
| Male | 291 (40.08) | 570 (42.57) |  |  |
| Female | 435 (59.92) | 769 (57.43) |  |  |
| Race, *n(%)* |  |  | χ²=7.31 | **0.026** |
| Han ethnic group | 666 (91.74) | 1177 (87.90) |  |  |
| National minority | 4 (0.55) | 13 (0.97) |  |  |
| Unknown | 56 (7.71) | 149 (11.13) |  |  |
| **Vital signs** |  |  |  |  |
| Temperature, ℃ | 36.65 ± 0.80 | 36.66 ± 0.80 | t=-0.15 | 0.880 |
| Heart rate, times/min | 93.78 ± 41.51 | 93.92 ± 34.50 | t=-0.08 | 0.933 |
| SBP, mmhg | 140.47 ± 36.01 | 139.73 ± 35.81 | t=0.45 | 0.656 |
| DBP, mmhg | 82.30 ± 33.18 | 81.67 ± 28.16 | t=0.45 | 0.651 |
| MBP, mmhg | 101.69 ± 29.68 | 101.03 ± 27.25 | t=0.51 | 0.609 |
| **Laboratory values on admission** |  |  |  |  |
| PH | 7.41 (7.36, 7.46) | 7.40 (7.36, 7.44) | Z=-2.42 | **0.016** |
| PaO_2_, mmhg | 85.45 (64.40, 119.80) | 90.40 (69.15, 114.46) | Z=-1.50 | 0.135 |
| PaCO_2_, mmhg | 36.10 (31.02, 42.09) | 36.20 (31.30, 42.10) | Z=-0.59 | 0.556 |
| Lac, mmol/l | 2.16 (1.30, 3.35) | 2.19 (1.40, 3.40) | Z=-0.93 | 0.354 |
| HsCRP, mg/L | 11.14 (1.39, 47.01) | 9.46 (1.05, 49.53) | Z=-0.74 | 0.459 |
| WBC, ×10^9^ | 9.04 (6.71, 12.30) | 9.35 (6.78, 12.66) | Z=-1.19 | 0.236 |
| PCT, ng/ml | 0.15 (0.06, 0.76) | 0.15 (0.06, 0.77) | Z=-0.27 | 0.784 |
| Tbil,umol/l | 17.85 (12.20, 26.70) | 17.20 (12.00, 24.40) | Z=-1.57 | 0.115 |
| ALT,U/L | 26.00 (19.00, 41.00) | 25.00 (19.00, 39.00) | Z=-1.47 | 0.141 |
| AST,U/L | 25.00 (18.00, 40.00) | 28.00 (21.00, 42.00) | Z=-3.37 | **<.001** |
| Creatinine, mg/dL | 81.70 (60.40, 128.52) | 82.81 (60.60, 128.30) | Z=-0.11 | 0.915 |
| ALB, g/L | 36.50 (31.60, 41.00) | 37.00 (31.90, 41.30) | Z=-0.81 | 0.417 |
| BUN,mg/dL | 7.70 (5.70, 11.90) | 7.60 (5.50, 12.00) | Z=-0.67 | 0.506 |
| HB, g/L | 122.00 (102.00, 139.30) | 123.00 (102.65, 140.00) | Z=-0.74 | 0.457 |
| PLT, ×10^9^ | 195.00 (145.25, 250.75) | 197.00 (146.00, 252.00) | Z=-0.30 | 0.763 |
| PT,s | 14.40 (13.60, 15.50) | 14.40 (13.50, 15.50) | Z=-0.36 | 0.716 |
| APTT, s | 36.00 (32.70, 39.90) | 35.90 (32.40, 39.81) | Z=-0.92 | 0.358 |
| FIB, g/L | 3.63 (2.87, 4.71) | 3.59 (2.86, 4.75) | Z=-0.26 | 0.793 |
| DD, mg/L | 2.84 (0.88, 65.60) | 2.96 (0.91, 69.98) | Z=-0.26 | 0.796 |
| **Final diagnosis, *n* (%)** |  |  |  |  |
| Pneumonia | 141 (19.42) | 264 (19.72) | χ²=0.03 | 0.872 |
| Asthma | 74 (10.19) | 121 (9.04) | χ²=0.74 | 0.391 |
| Pleural effusion | 73 (10.06) | 122 (9.11) | χ²=0.49 | 0.484 |
| Bronchitis | 49 (6.75) | 119 (8.89) | χ²=2.88 | 0.090 |
| COPD | 66 (9.09) | 102 (7.62) | χ²=1.37 | 0.242 |
| Pulmonary embolism | 32 (4.41) | 57 (4.26) | χ²=0.03 | 0.872 |
| Pulmonary fibrosis | 37 (5.10) | 56 (4.18) | χ²=0.91 | 0.339 |
| Lung cancer | 34 (4.68) | 51 (3.81) | χ²=0.91 | 0.340 |
| Cardiac insufficiency | 33 (4.55) | 87 (6.50) | χ²=3.28 | 0.070 |
| Myocardial infarction | 25 (3.44) | 38 (2.84) | χ²=0.58 | 0.445 |
| Aortic dissection | 20 (2.75) | 32 (2.39) | χ²=0.26 | 0.613 |
| Atrial fibrillation | 31 (4.27) | 58 (4.33) | χ²=0.00 | 0.948 |
| Hysteria | 31 (4.27) | 58 (4.33) | χ²=0.00 | 0.948 |
| Poisoning | 31 (4.27) | 56 (4.18) | χ²=0.01 | 0.924 |
| Trauma | 17 (2.34) | 26 (1.94) | χ²=0.37 | 0.543 |
| Other diseases | 72 (9.92) | 111 (8.29) | χ²=1.54 | 0.214 |
| **Comorbidites, *n* (%)** |  |  |  |  |
| Hypertensive | 399 (54.96) | 708 (52.88) | χ²=0.82 | 0.365 |
| Diabetes | 209 (28.79) | 393 (29.35) | χ²=0.07 | 0.788 |
| Coronary heart disease | 108 (14.88) | 183 (13.67) | χ²=0.57 | 0.451 |
| Cerebral infarction | 176 (24.24) | 308 (23.00) | χ²=0.40 | 0.525 |
| Alzheimers disease | 44 (6.06) | 67 (5.00) | χ²=1.03 | 0.309 |
| Cirrhosis | 31 (4.27) | 42 (3.14) | χ²=1.77 | 0.183 |
| Renal insufficiency | 40 (5.51) | 69 (5.15) | χ²=0.12 | 0.729 |
| Malignant tumour | 93 (12.81) | 147 (10.98) | χ²=1.54 | 0.215 |
| **Outcome** |  |  |  |  |
| 7-day Intubation |  |  | χ²=0.05 | 0.818 |
| No | 594 (81.82) | 1101 (82.23) |  |  |
| Yes | 132 (18.18) | 238 (17.77) |  |  |
| 28-day Mortality |  |  | χ²=2.24 | 0.134 |
| No | 582 (80.17) | 1109 (82.82) |  |  |
| Yes | 144 (19.83) | 230 (17.18) |  |  |

Table S11. Baseline characteristics of patients

| Variables | G1 (n = 681) | G2 (n = 685) | G3 (n = 699) | Statistic | *P* |
| --- | --- | --- | --- | --- | --- |
|  |  |  |  |  |  |
| SR | 3.34 ± 0.68 | 4.81 ± 0.28 | 6.09 ± 0.74 | F=3604.15 | **<.001** |
| **Characteristic** |  |  |  |  |  |
| Age, years | 73.32 ± 15.58 | 71.91 ± 16.69 | 68.32 ± 16.90 | F=17.09 | **<.001** |
| BMI | 22.69 ± 4.59 | 22.41 ± 4.66 | 22.50 ± 4.40 | F=0.66 | 0.517 |
| Sex, *n(%)* |  |  |  | χ²=1.29 | 0.525 |
| Male | 294 (43.17) | 275 (40.15) | 292 (41.77) |  |  |
| Female | 387 (56.83) | 410 (59.85) | 407 (58.23) |  |  |
| Race, *n(%)* |  |  |  | χ²=1.52 | 0.823 |
| Han ethnic group | 612 (89.87) | 606 (88.47) | 625 (89.41) |  |  |
| National minority | 6 (0.88) | 7 (1.02) | 4 (0.57) |  |  |
| Unknown | 63 (9.25) | 72 (10.51) | 70 (10.01) |  |  |
| **Vital signs** |  |  |  |  |  |
| Temperature, ℃ | 36.74 ± 0.84 | 36.66 ± 0.85 | 36.57 ± 0.69 | F=7.85 | **<.001** |
| Heart rate, times/min | 102.56 ± 54.98 | 92.26 ± 22.66 | 86.99 ± 22.35 | F=32.27 | **<.001** |
| SBP, mmhg | 140.42 ± 34.83 | 139.14 ± 37.78 | 140.40 ± 34.98 | F=0.29 | 0.751 |
| DBP, mmhg | 82.32 ± 22.66 | 80.01 ± 20.35 | 83.33 ± 41.87 | F=2.22 | 0.109 |
| MBP, mmhg | 101.69 ± 25.03 | 99.72 ± 24.88 | 102.35 ± 33.44 | F=1.63 | 0.196 |
| **Laboratory values on admission** |  |  |  |  |  |
| PH | 7.40 (7.35,7.45) | 7.41 (7.36,7.46) | 7.41 (7.36,7.45) | χ²=2.85# | 0.241 |
| PaO_2_, mmhg | 90.70 (67.30,118.00) | 89.40 (68.50,119.20) | 86.60 (64.70,111.00) | χ²=5.14# | 0.077 |
| PaCO_2_, mmhg | 36.90 (30.98,45.75) | 35.70 (30.80,41.40) | 36.10 (31.51,40.70) | χ²=10.49# | **0.005** |
| Lac, mmol/l | 2.30 (1.40,4.10) | 2.04 (1.30,3.30) | 2.16 (1.40,3.15) | χ²=13.93# | **<.001** |
| HsCRP, mg/L | 21.38 (3.65,73.78) | 9.44 (0.90,46.51) | 3.59 (0.71,24.42) | χ²=106.60# | **<.001** |
| WBC, ×10^9^ | 10.37 (7.36,13.63) | 9.10 (6.90,12.35) | 8.29 (6.17,11.29) | χ²=50.90# | **<.001** |
| PCT, ng/ml | 0.21 (0.07,1.06) | 0.16 (0.06,0.71) | 0.11 (0.05,0.58) | χ²=39.53# | **<.001** |
| Tbil,umol/l | 17.00 (11.30,26.00) | 17.00 (11.70,23.66) | 18.00 (13.90,26.33) | χ²=10.31# | **0.006** |
| ALT,U/L | 27.00 (20.00,44.00) | 26.00 (18.00,41.00) | 24.20 (19.00,36.05) | χ²=13.98# | **<.001** |
| AST,U/L | 30.00 (23.00,49.00) | 25.00 (20.00,39.00) | 25.80 (19.00,38.70) | χ²=38.60# | **<.001** |
| Creatinine, mg/dL | 87.80 (59.00,146.90) | 82.70 (59.70,111.20) | 79.50 (61.45,126.40) | χ²=10.13# | **0.006** |
| ALB, g/L | 36.11 (31.00,40.50) | 36.70 (31.40,41.10) | 37.70 (33.29,42.10) | χ²=17.74# | **<.001** |
| BUN,mg/dL | 8.20 (6.09,13.60) | 7.50 (5.70,11.30) | 7.20 (5.10,11.03) | χ²=29.89# | **<.001** |
| HB, g/L | 121.00 (100.00,137.00) | 122.00 (102.00,138.00) | 126.00 (107.00,143.00) | χ²=15.50# | **<.001** |
| PLT, ×10^9^ | 195.00 (146.00,256.00) | 198.20 (147.00,247.00) | 195.00 (145.00,252.00) | χ²=0.48# | 0.788 |
| PT,s | 14.60 (13.70,15.90) | 14.40 (13.60,15.40) | 14.10 (13.40,15.10) | χ²=33.75# | **<.001** |
| APTT, s | 36.40 (33.20,40.10) | 36.00 (32.28,40.50) | 35.40 (31.90,38.56) | χ²=23.77# | **<.001** |
| FIB, g/L | 4.07 (3.17,5.30) | 3.67 (2.82,4.61) | 3.24 (2.69,4.19) | χ²=94.53# | **<.001** |
| DD, mg/L | 4.54 (1.37,87.68) | 2.77 (0.88,60.14) | 2.29 (0.50,51.82) | χ²=38.58# | **<.001** |
| **Final diagnosis, *n* (%)** |  |  |  |  |  |
| Pneumonia | 127 (18.65) | 129 (18.83) | 149 (21.32) | χ²=1.95 | 0.377 |
| Asthma | 68 (9.99) | 66 (9.64) | 61 (8.73) | χ²=0.68 | 0.711 |
| Pleural effusion | 72 (10.57) | 59 (8.61) | 64 (9.16) | χ²=1.64 | 0.441 |
| Bronchitis | 71 (10.43) | 66 (9.64) | 31 (4.43) | χ²=19.65 | **<.001** |
| COPD | 74 (10.87) | 47 (6.86) | 47 (6.72) | χ²=10.15 | **0.006** |
| Pulmonary embolism | 27 (3.96) | 25 (3.65) | 37 (5.29) | χ²=2.56 | 0.278 |
| Pulmonary fibrosis | 21 (3.08) | 27 (3.94) | 45 (6.44) | χ²=9.78 | **0.008** |
| Lung cancer | 26 (3.82) | 34 (4.96) | 25 (3.58) | χ²=1.92 | 0.384 |
| Cardiac insufficiency | 37 (5.43) | 40 (5.84) | 43 (6.15) | χ²=0.33 | 0.849 |
| Myocardial infarction | 26 (3.82) | 25 (3.65) | 12 (1.72) | χ²=6.39 | **0.041** |
| Aortic dissection | 12 (1.76) | 19 (2.77) | 21 (3.00) | χ²=2.44 | 0.295 |
| Atrial fibrillation | 35 (5.14) | 24 (3.50) | 30 (4.29) | χ²=2.22 | 0.330 |
| Hysteria | 27 (3.96) | 32 (4.67) | 30 (4.29) | χ²=0.41 | 0.813 |
| Poisoning | 27 (3.96) | 35 (5.11) | 25 (3.58) | χ²=2.17 | 0.338 |
| Trauma | 6 (0.88) | 14 (2.04) | 23 (3.29) | χ²=9.83 | **0.007** |
| Other diseases | 47 (6.90) | 62 (9.05) | 74 (10.59) | χ²=5.84 | 0.054 |
| **Comorbidites, *n* (%)** |  |  |  |  |  |
| Hypertensive | 396 (58.15) | 371 (54.16) | 340 (48.64) | χ²=12.67 | **0.002** |
| Diabetes | 220 (32.31) | 180 (26.28) | 202 (28.90) | χ²=6.04 | **0.049** |
| Coronary heart disease | 110 (16.15) | 75 (10.95) | 106 (15.16) | χ²=8.64 | **0.013** |
| Cerebral infarction | 185 (27.17) | 165 (24.09) | 134 (19.17) | χ²=12.53 | **0.002** |
| Alzheimers disease | 44 (6.46) | 41 (5.99) | 26 (3.72) | χ²=5.85 | 0.054 |
| Cirrhosis | 31 (4.55) | 21 (3.07) | 21 (3.00) | χ²=3.09 | 0.214 |
| Renal insufficiency | 45 (6.61) | 32 (4.67) | 32 (4.58) | χ²=3.60 | 0.165 |
| Malignant tumour | 112 (16.45) | 68 (9.93) | 60 (8.58) | χ²=23.63 | **<.001** |
| **Outcome** |  |  |  |  |  |
| 7-day Intubation |  |  |  | χ²=11.49 | **0.003** |
| No | 346 (77.23) | 377 (84.72) | 378 (84.75) |  |  |
| Yes | 102 (22.77) | 68 (15.28) | 68 (15.25) |  |  |
| 28-day Mortality |  |  |  | χ²=27.33 | **<.001** |
| No | 338 (75.45) | 378 (84.94) | 393 (88.12) |  |  |
| Yes | 110 (24.55) | 67 (15.06) | 53 (11.88) |  |  |

### Table S12. Association between SR and 7-day intubation

| Variables | Model1 | |  | Model2 | |  | Model3 | |
| --- | --- | --- | --- | --- | --- | --- | --- | --- |
|  | HR (95%CI) | *P* |  | HR (95%CI) | *P* |  | HR (95%CI) | *P* |
| SR | 0.87 (0.80 ~ 0.94) | **<.001** |  | 0.85 (0.78 ~ 0.92) | **<.001** |  | 0.85 (0.78 ~ 0.93) | **<.001** |
| SR group |  |  |  |  |  |  |  |  |
| 1 | 1.00 (Reference) |  |  | 1.00 (Reference) |  |  | 1.00 (Reference) |  |
| 2 | 0.66 (0.52 ~ 0.85) | **0.001** |  | 0.61 (0.48 ~ 0.79) | **<.001** |  | 0.62 (0.48 ~ 0.80) | **<.001** |
| 3 | 0.69 (0.54 ~ 0.88) | **0.003** |  | 0.66 (0.52 ~ 0.85) | **0.001** |  | 0.67 (0.52 ~ 0.86) | **0.002** |
| HR: hazard ratio; CI: confidence interval  Model 1: Crude  Model 2: Adjusted for sex, race, age, and BMI  Model 3: Adjusted for sex, race, diabetes, cerebral infarction, alzheimers disease,atrial fibrillation,HR, Lac, PaCO_2_,hsCRP, PCT,AST,Cr,APTT and FIB | | | | | | | | |

Table S13.Univariate COX regression for7-day intubation

| Variables | β | S.E | Z | *P* | HR (95%CI) |
| --- | --- | --- | --- | --- | --- |
|  |  |  |  |  |  |
| Sex |  |  |  |  |  |
| Male |  |  |  |  | 1.00 (Reference) |
| Female | 0.35 | 0.11 | 3.17 | **0.002** | 1.42 (1.14 ~ 1.75) |
| Race |  |  |  |  |  |
| Han ethnic group |  |  |  |  | 1.00 (Reference) |
| National minority | 0.27 | 0.50 | 0.53 | 0.594 | 1.31 (0.49 ~ 3.51) |
| UNKNOWN | 0.55 | 0.15 | 3.80 | **<.001** | 1.74 (1.31 ~ 2.31) |
| Hypertensive |  |  |  |  |  |
| No |  |  |  |  | 1.00 (Reference) |
| YES | 0.07 | 0.10 | 0.69 | 0.493 | 1.07 (0.88 ~ 1.32) |
| Diabetes |  |  |  |  |  |
| No |  |  |  |  | 1.00 (Reference) |
| YES | 0.46 | 0.11 | 4.26 | **<.001** | 1.58 (1.28 ~ 1.94) |
| Coronary heart disease |  |  |  |  |  |
| No |  |  |  |  | 1.00 (Reference) |
| YES | -0.24 | 0.16 | -1.50 | 0.135 | 0.78 (0.57 ~ 1.08) |
| Cerebral infarction |  |  |  |  |  |
| No |  |  |  |  | 1.00 (Reference) |
| YES | -0.30 | 0.13 | -2.24 | **0.025** | 0.74 (0.57 ~ 0.96) |
| Alzheimers disease |  |  |  |  |  |
| No |  |  |  |  | 1.00 (Reference) |
| YES | 0.41 | 0.19 | 2.12 | **0.034** | 1.51 (1.03 ~ 2.20) |
| Cirrhosis |  |  |  |  |  |
| No |  |  |  |  | 1.00 (Reference) |
| YES | -0.08 | 0.29 | -0.28 | 0.782 | 0.92 (0.52 ~ 1.64) |
| Renal insufficiency |  |  |  |  |  |
| No |  |  |  |  | 1.00 (Reference) |
| YES | 0.27 | 0.21 | 1.30 | 0.194 | 1.31 (0.87 ~ 1.96) |
| Malignant tumour |  |  |  |  |  |
| No |  |  |  |  | 1.00 (Reference) |
| YES | 0.19 | 0.15 | 1.24 | 0.215 | 1.21 (0.90 ~ 1.63) |
| Atrial fibrillation |  |  |  |  |  |
| No |  |  |  |  | 1.00 (Reference) |
| YES | -0.89 | 0.38 | -2.32 | **0.020** | 0.41 (0.20 ~ 0.87) |
| Cardiac insufficiency |  |  |  |  |  |
| No |  |  |  |  | 1.00 (Reference) |
| YES | -0.22 | 0.24 | -0.90 | 0.368 | 0.80 (0.50 ~ 1.29) |
| Bronchitis |  |  |  |  |  |
| No |  |  |  |  | 1.00 (Reference) |
| YES | -0.08 | 0.20 | -0.40 | 0.690 | 0.92 (0.63 ~ 1.36) |
| COPD |  |  |  |  |  |
| No |  |  |  |  | 1.00 (Reference) |
| YES | 0.25 | 0.17 | 1.45 | 0.148 | 1.28 (0.91 ~ 1.80) |
| BMI | -0.04 | 0.75 | -0.05 | 0.961 | 0.96 (0.22 ~ 4.19) |
| Age | 0.00 | 0.00 | 0.43 | 0.665 | 1.00 (1.00 ~ 1.01) |
| Heart rate | 0.01 | 0.00 | 4.64 | **<.001** | 1.01 (1.01 ~ 1.01) |
| SBP | -0.00 | 0.00 | -0.75 | 0.453 | 1.00 (1.00 ~ 1.00) |
| DBP | -0.00 | 0.00 | -1.45 | 0.147 | 1.00 (0.99 ~ 1.00) |
| MBP | -0.00 | 0.00 | -1.31 | 0.189 | 1.00 (0.99 ~ 1.00) |
| Temperature | 0.02 | 0.06 | 0.38 | 0.702 | 1.02 (0.90 ~ 1.16) |
| PH | -0.61 | 0.45 | -1.37 | 0.172 | 0.54 (0.22 ~ 1.31) |
| PaO_2_ | -0.00 | 0.00 | -1.18 | 0.237 | 1.00 (1.00 ~ 1.00) |
| PaCO_2_ | 0.01 | 0.00 | 2.25 | **0.024** | 1.01 (1.01 ~ 1.01) |
| Lac | 0.05 | 0.01 | 3.35 | **<.001** | 1.05 (1.02 ~ 1.08) |
| HsCRP | 0.01 | 0.00 | 2.43 | **0.015** | 1.01 (1.01 ~ 1.01) |
| WBC | 0.00 | 0.01 | 0.83 | 0.404 | 1.00 (0.99 ~ 1.01) |
| PCT | 0.01 | 0.00 | 2.23 | **0.026** | 1.01 (1.01 ~ 1.02) |
| Tbil | 0.00 | 0.00 | 0.34 | 0.733 | 1.00 (1.00 ~ 1.00) |
| ALT | 0.00 | 0.00 | 1.09 | 0.276 | 1.00 (1.00 ~ 1.00) |
| AST | 0.01 | 0.00 | 1.99 | **0.047** | 1.01 (1.01 ~ 1.01) |
| Cr | 0.01 | 0.00 | 2.86 | **0.004** | 1.01 (1.01 ~ 1.01) |
| ALB | -0.00 | 0.01 | -0.33 | 0.742 | 1.00 (0.98 ~ 1.01) |
| BUN | 0.00 | 0.00 | 0.26 | 0.795 | 1.00 (0.99 ~ 1.01) |
| HB | 0.00 | 0.00 | 1.29 | 0.198 | 1.00 (1.00 ~ 1.01) |
| PLT | 0.00 | 0.00 | 0.09 | 0.927 | 1.00 (1.00 ~ 1.00) |
| PT | 0.00 | 0.01 | 0.23 | 0.817 | 1.00 (0.98 ~ 1.03) |
| APTT | 0.01 | 0.00 | 2.07 | **0.039** | 1.01 (1.01 ~ 1.01) |
| FIB | 0.04 | 0.01 | 2.40 | **0.017** | 1.04 (1.01 ~ 1.07) |
| DD | 0.00 | 0.00 | 0.43 | 0.668 | 1.00 (1.00 ~ 1.00) |

| 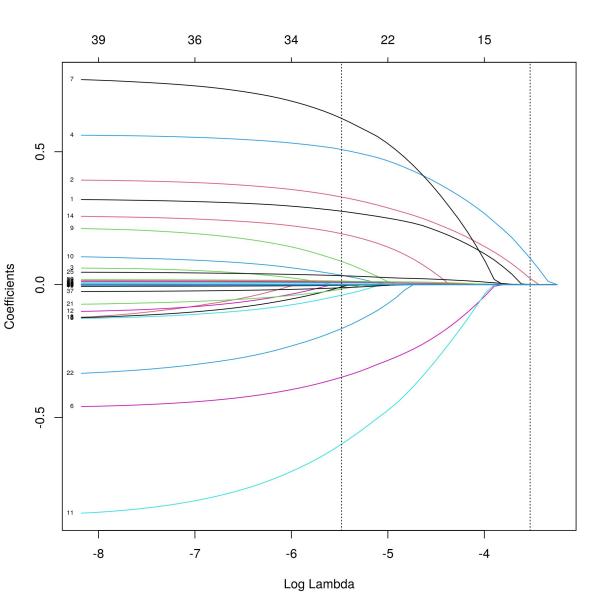  A | 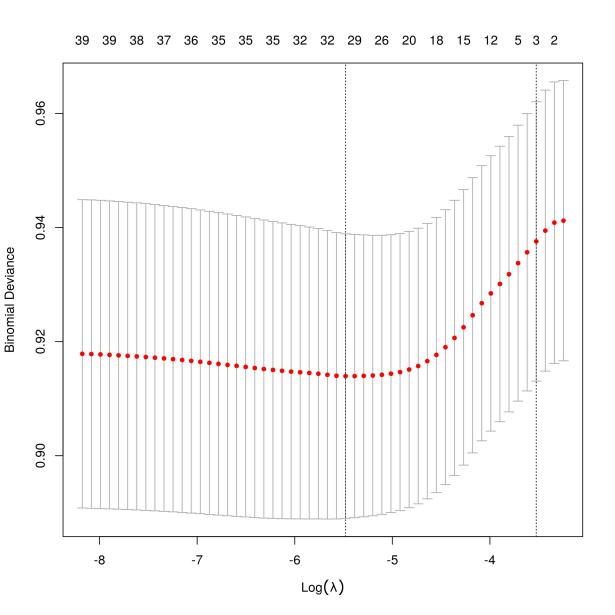  B |
| --- | --- |

Fig. S8 Results of the factor selection. A Plot of the LASSO coefficient profiles. B Tuning parameter (λ) selection cross-validation error curve.

### Table S14. Association between SR and 28-day mortality

| Variables | Model1 | |  | Model2 | |  | Model3 | |
| --- | --- | --- | --- | --- | --- | --- | --- | --- |
|  | HR (95%CI) | *P* |  | HR (95%CI) | *P* |  | HR (95%CI) | *P* |
| SR | 0.73 (0.68 ~ 0.79) | **<.001** |  | 0.74 (0.68 ~ 0.81) | **<.001** |  | 0.77 (0.71 ~ 0.84) | **<.001** |
| SR group |  |  |  |  |  |  |  |  |
| 1 | 1.00 (Reference) |  |  | 1.00 (Reference) |  |  | 1.00 (Reference) |  |
| 2 | 0.59 (0.46 ~ 0.75) | **<.001** |  | 0.60 (0.47 ~ 0.77) | **<.001** |  | 0.66 (0.51 ~ 0.85) | **0.001** |
| 3 | 0.49 (0.38 ~ 0.64) | **<.001** |  | 0.52 (0.40 ~ 0.68) | **<.001** |  | 0.59 (0.45 ~ 0.77) | **<.001** |
| HR: hazard ratio; CI: confidence interval  Model 1: Crude  Model 2: Adjusted for sex, race, age, and BMI  Model 3: Adjusted for age, malignant tumor, SBP, Lac, hsCRP, WBC, ALB, BUN, Hb, PT | | | | | | | | |

Table S15.Univariate COX regression for 28-day mortality

| Variables | β | S.E | Z | *P* | HR (95%CI) |
| --- | --- | --- | --- | --- | --- |
|  |  |  |  |  |  |
| Sex |  |  |  |  |  |
| Male |  |  |  |  | 1.00 (Reference) |
| Female | 0.14 | 0.11 | 1.33 | 0.182 | 1.15 (0.94 ~ 1.42) |
| Race |  |  |  |  |  |
| Han ethnic group |  |  |  |  | 1.00 (Reference) |
| National minority | 0.28 | 0.50 | 0.55 | 0.580 | 1.32 (0.49 ~ 3.54) |
| UNKNOWN | 0.18 | 0.16 | 1.08 | 0.280 | 1.19 (0.87 ~ 1.65) |
| Hypertensive |  |  |  |  |  |
| No |  |  |  |  | 1.00 (Reference) |
| YES | 0.16 | 0.10 | 1.52 | 0.129 | 1.17 (0.95 ~ 1.44) |
| Diabetes |  |  |  |  |  |
| No |  |  |  |  | 1.00 (Reference) |
| YES | 0.03 | 0.11 | 0.26 | 0.793 | 1.03 (0.83 ~ 1.29) |
| Coronary heart disease |  |  |  |  |  |
| No |  |  |  |  | 1.00 (Reference) |
| YES | -0.23 | 0.16 | -1.41 | 0.158 | 0.80 (0.58 ~ 1.09) |
| Cerebral infarction |  |  |  |  |  |
| No |  |  |  |  | 1.00 (Reference) |
| YES | 0.19 | 0.12 | 1.67 | 0.095 | 1.21 (0.97 ~ 1.53) |
| Alzheimers disease |  |  |  |  |  |
| No |  |  |  |  | 1.00 (Reference) |
| YES | 0.12 | 0.22 | 0.54 | 0.590 | 1.13 (0.73 ~ 1.73) |
| Cirrhosis |  |  |  |  |  |
| No |  |  |  |  | 1.00 (Reference) |
| YES | 0.52 | 0.23 | 2.28 | **0.023** | 1.69 (1.08 ~ 2.65) |
| Renal insufficiency |  |  |  |  |  |
| No |  |  |  |  | 1.00 (Reference) |
| YES | -0.10 | 0.24 | -0.43 | 0.665 | 0.90 (0.56 ~ 1.45) |
| Malignant tumour |  |  |  |  |  |
| No |  |  |  |  | 1.00 (Reference) |
| YES | 0.93 | 0.12 | 7.54 | **<.001** | 2.53 (1.99 ~ 3.22) |
| Atrial fibrillation |  |  |  |  |  |
| No |  |  |  |  | 1.00 (Reference) |
| YES | 0.11 | 0.24 | 0.47 | 0.641 | 1.12 (0.70 ~ 1.80) |
| Cardiac insufficiency |  |  |  |  |  |
| No |  |  |  |  | 1.00 (Reference) |
| YES | 0.00 | 0.22 | 0.01 | 0.994 | 1.00 (0.65 ~ 1.54) |
| Bronchitis |  |  |  |  |  |
| No |  |  |  |  | 1.00 (Reference) |
| YES | 0.16 | 0.18 | 0.90 | 0.369 | 1.17 (0.83 ~ 1.66) |
| COPD |  |  |  |  |  |
| No |  |  |  |  | 1.00 (Reference) |
| YES | 0.05 | 0.18 | 0.29 | 0.774 | 1.05 (0.73 ~ 1.52) |
| BMI | -17.40 | 4466.16 | -0.00 | 0.997 | 0.00 (0.00 ~ Inf) |
| Age | 0.02 | 0.00 | 5.85 | **<.001** | 1.02 (1.01 ~ 1.03) |
| Heart rate | 0.01 | 0.00 | 2.05 | **0.040** | 1.01 (1.01 ~ 1.01) |
| SBP | -0.01 | 0.00 | -7.57 | **<.001** | 0.99 (0.98 ~ 0.99) |
| DBP | -0.02 | 0.00 | -5.98 | **<.001** | 0.98 (0.98 ~ 0.99) |
| MBP | -0.02 | 0.00 | -7.17 | **<.001** | 0.98 (0.98 ~ 0.99) |
| Temperature | -0.06 | 0.07 | -0.90 | 0.370 | 0.94 (0.82 ~ 1.08) |
| PH | -1.08 | 0.42 | -2.54 | **0.011** | 0.34 (0.15 ~ 0.78) |
| PaO_2_ | 0.00 | 0.00 | 0.96 | 0.335 | 1.00 (1.00 ~ 1.00) |
| PaCO_2_ | -0.01 | 0.00 | -2.71 | **0.007** | 0.99 (0.98 ~ 0.99) |
| Lac | 0.09 | 0.01 | 8.73 | **<.001** | 1.10 (1.08 ~ 1.12) |
| HsCRP | 0.01 | 0.00 | 9.72 | **<.001** | 1.01 (1.01 ~ 1.01) |
| WBC | 0.02 | 0.00 | 6.29 | **<.001** | 1.02 (1.01 ~ 1.02) |
| PCT | 0.02 | 0.00 | 5.79 | **<.001** | 1.02 (1.01 ~ 1.03) |
| Tbil | 0.01 | 0.00 | 5.57 | **<.001** | 1.01 (1.01 ~ 1.01) |
| ALT | 0.01 | 0.00 | 4.68 | **<.001** | 1.01 (1.01 ~ 1.01) |
| AST | 0.01 | 0.00 | 3.10 | **0.002** | 1.01 (1.01 ~ 1.01) |
| Cr | 0.01 | 0.00 | 5.48 | **<.001** | 1.01 (1.01 ~ 1.01) |
| ALB | -0.07 | 0.01 | -9.83 | **<.001** | 0.93 (0.92 ~ 0.95) |
| BUN | 0.01 | 0.00 | 7.02 | **<.001** | 1.01 (1.01 ~ 1.01) |
| Hb | -0.01 | 0.00 | -8.17 | **<.001** | 0.99 (0.98 ~ 0.99) |
| PLT | 0.00 | 0.00 | 1.62 | 0.106 | 1.00 (1.00 ~ 1.00) |
| PT | 0.03 | 0.00 | 7.15 | **<.001** | 1.03 (1.02 ~ 1.04) |
| APTT | 0.01 | 0.00 | 3.14 | **0.002** | 1.01 (1.01 ~ 1.02) |
| FIB | 0.03 | 0.02 | 1.52 | 0.129 | 1.03 (0.99 ~ 1.06) |
| D-D | -0.01 | 0.00 | -2.71 | **0.007** | 0.99 (0.99 ~ 0.99) |

| 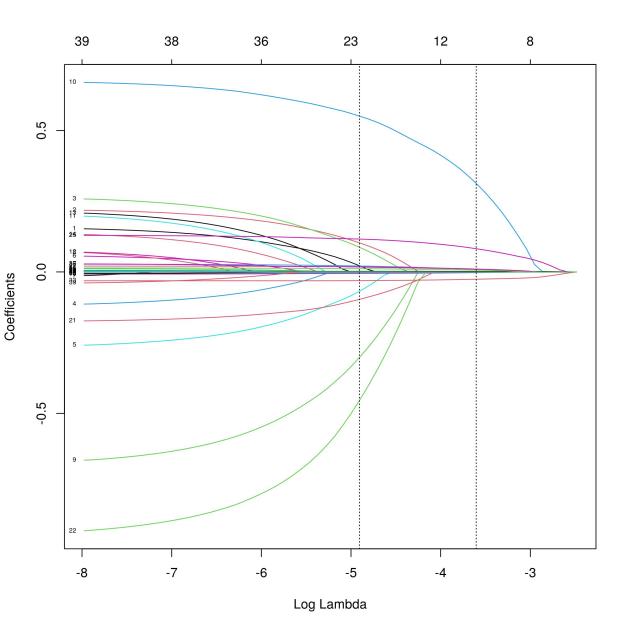  A | 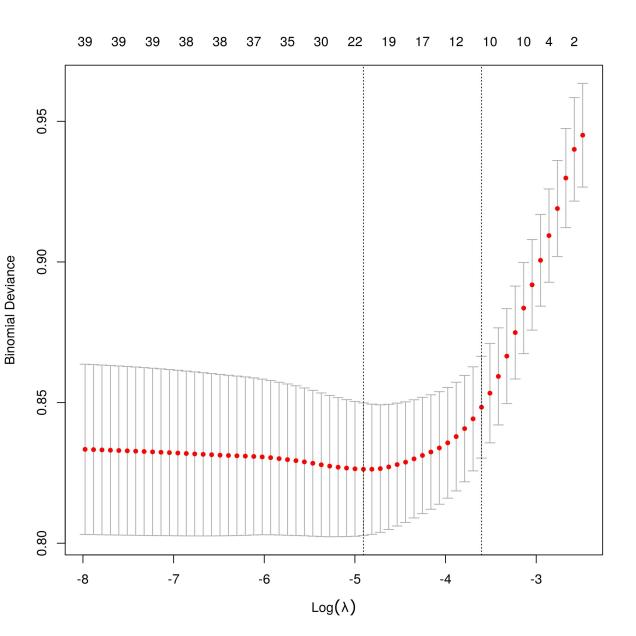  B |
| --- | --- |

Fig. S9 Results of the factor selection. A Plot of the LASSO coefficient profiles. B Tuning parameter (λ) selection cross-validation error curve.
